# Supplementary material for: Active ingredients and molecular targets of Taraxacum mongolicum against hepatocellular carcinoma: network pharmacology, molecular docking, and molecular dynamics simulation analysis
Source: PeerJ. 2022 Jul 18;10:e13737. doi: 10.7717/peerj.13737 (PMC9302432; doi:10.7717/peerj.13737)
Supplement: Supplemental Information 3 [file peerj-10-13737-s003.zip › Enrichment_GO/ColorByCluster.pdf]

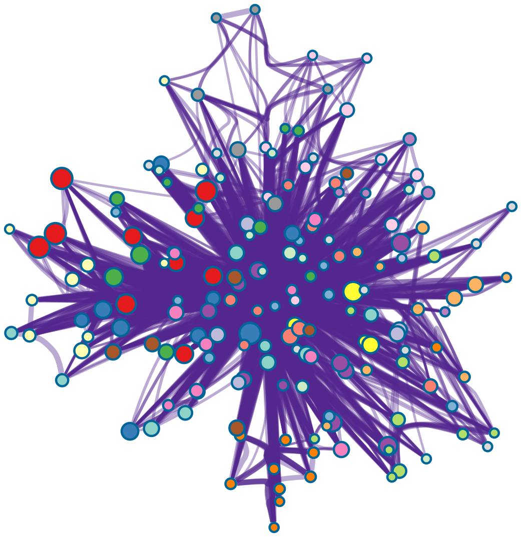

- gland development
- epithelial cell proliferation
- peptidyl-serine phosphorylation
- response to nutrient levels
- regulation of production of miRNAs involved in gene
- response to growth factor
- cellular response to abiotic stimulus
- glial cell differentiation
- mammary gland development
- regulation of DNA-binding transcription factor activity
- positive regulation of cellular protein localization
- rhythmic process
- regulation of proteolysis
- regulation of cyclin-dependent protein serine/threonin
- autophagy
- neuron death
- mammary gland alveolus development
- peptidyl-tyrosine autophosphorylation
- regulation of protein stability
- developmental growth

created by
http://metascape.org
