# Supplementary figures and images for: Active ingredients and molecular targets of Taraxacum mongolicum against hepatocellular carcinoma: network pharmacology, molecular docking, and molecular dynamics simulation analysis
Source: PeerJ. 2022 Jul 18;10:e13737. doi: 10.7717/peerj.13737 (PMC9302432; doi:10.7717/peerj.13737)

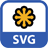

Supplement: Supplemental Information 3 [file peerj-10-13737-s003.zip › icon/SVG48.png]

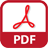

Supplement: Supplemental Information 3 [file peerj-10-13737-s003.zip › icon/PDF48.png]

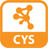

Supplement: Supplemental Information 3 [file peerj-10-13737-s003.zip › icon/CYS48.png]

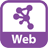

Supplement: Supplemental Information 3 [file peerj-10-13737-s003.zip › icon/WEB_CYS48.png]

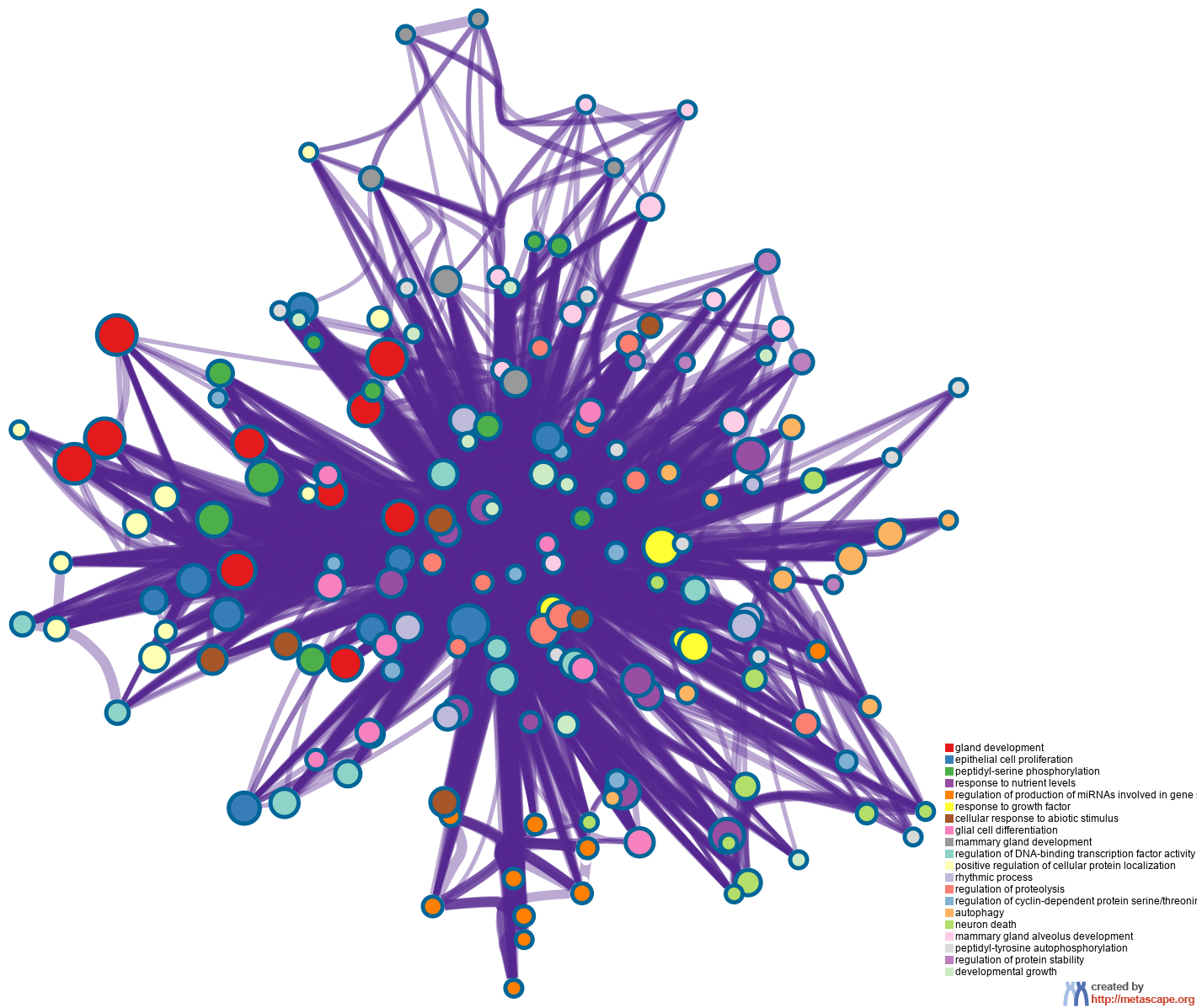

Supplement: Supplemental Information 3 [file peerj-10-13737-s003.zip › Enrichment_GO/ColorByCluster.png]

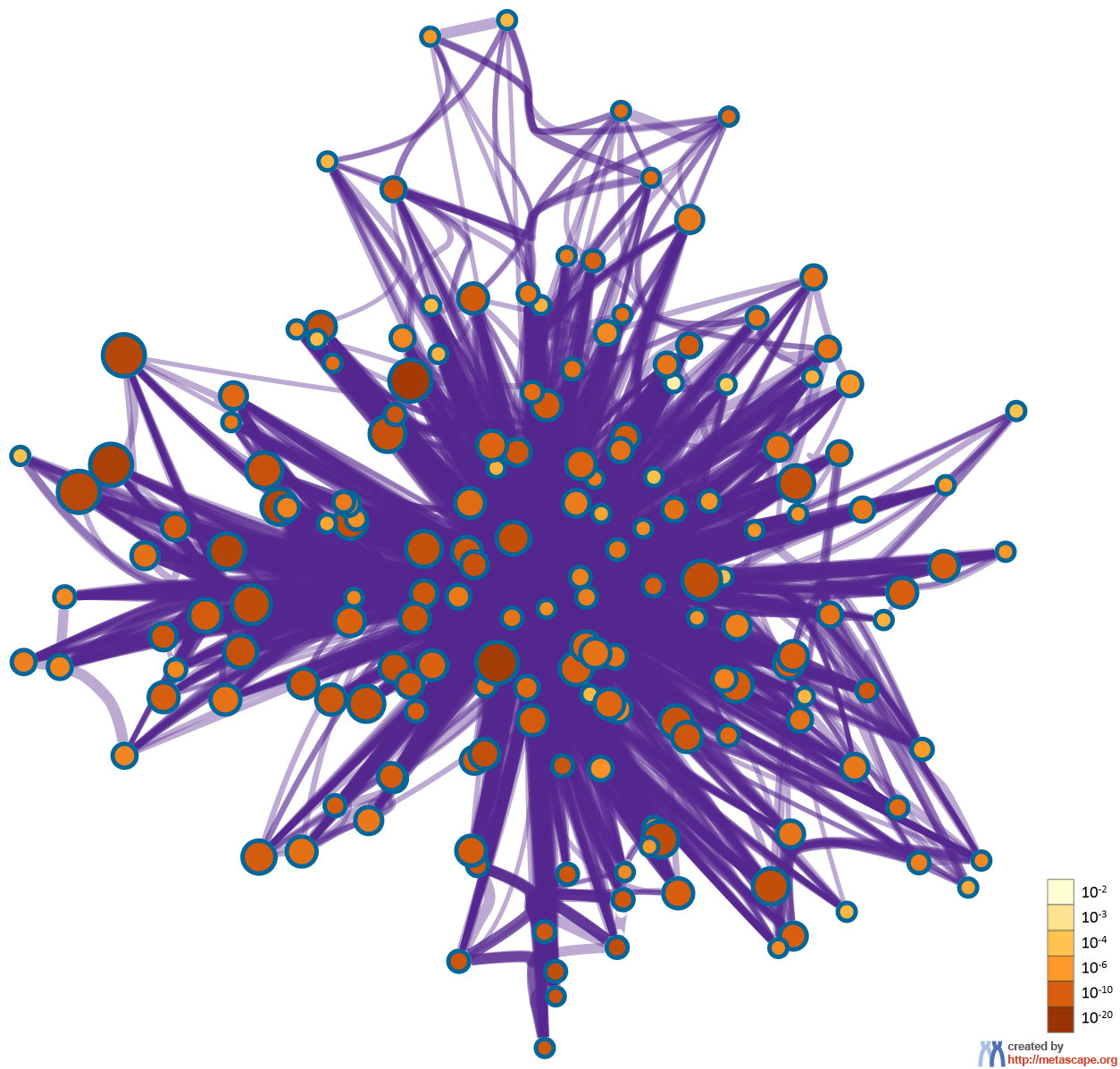

Supplement: Supplemental Information 3 [file peerj-10-13737-s003.zip › Enrichment_GO/ColorByPValue.png]

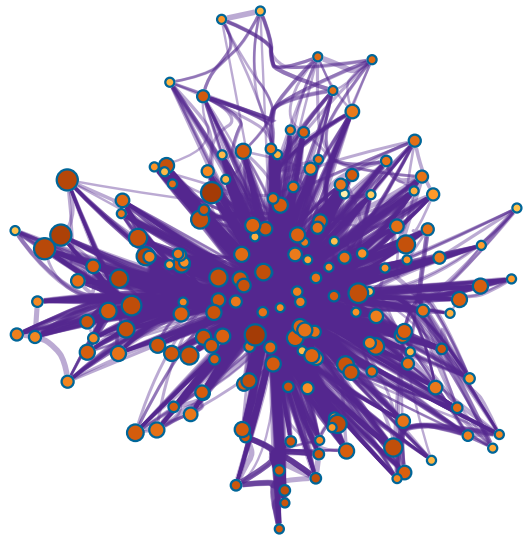

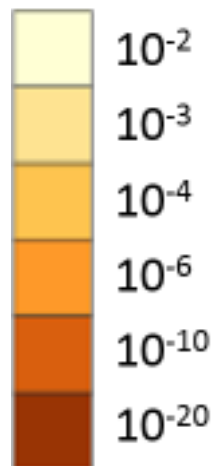

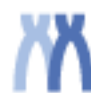 created by  
<http://metascape.org>

Supplement: Supplemental Information 3 [file peerj-10-13737-s003.zip › Enrichment_GO/ColorByPValue.pdf]

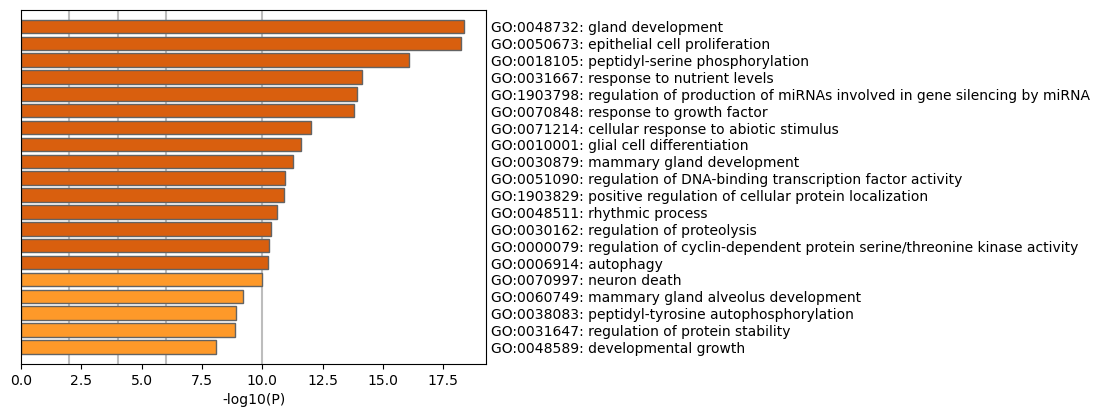

Supplement: Supplemental Information 3 [file peerj-10-13737-s003.zip › Enrichment_heatmap/HeatmapSelectedGO.png]

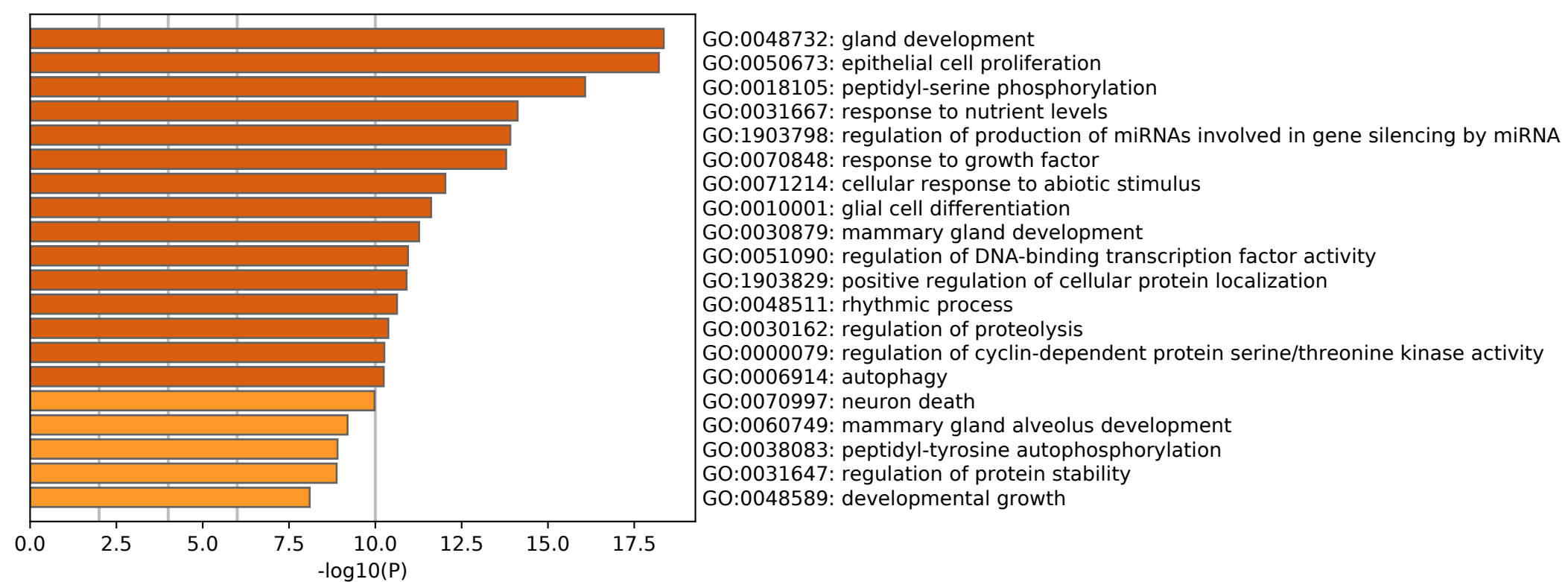

Supplement: Supplemental Information 3 [file peerj-10-13737-s003.zip › Enrichment_heatmap/HeatmapSelectedGO.pdf]

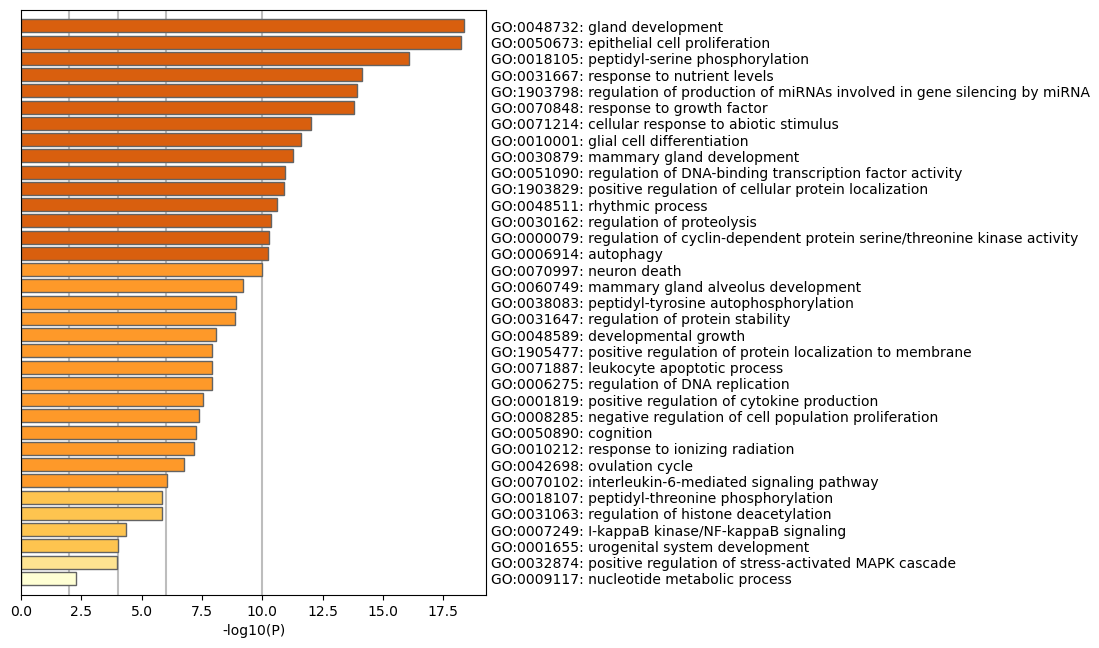

Supplement: Supplemental Information 3 [file peerj-10-13737-s003.zip › Enrichment_heatmap/HeatmapSelectedGOTop100.png]

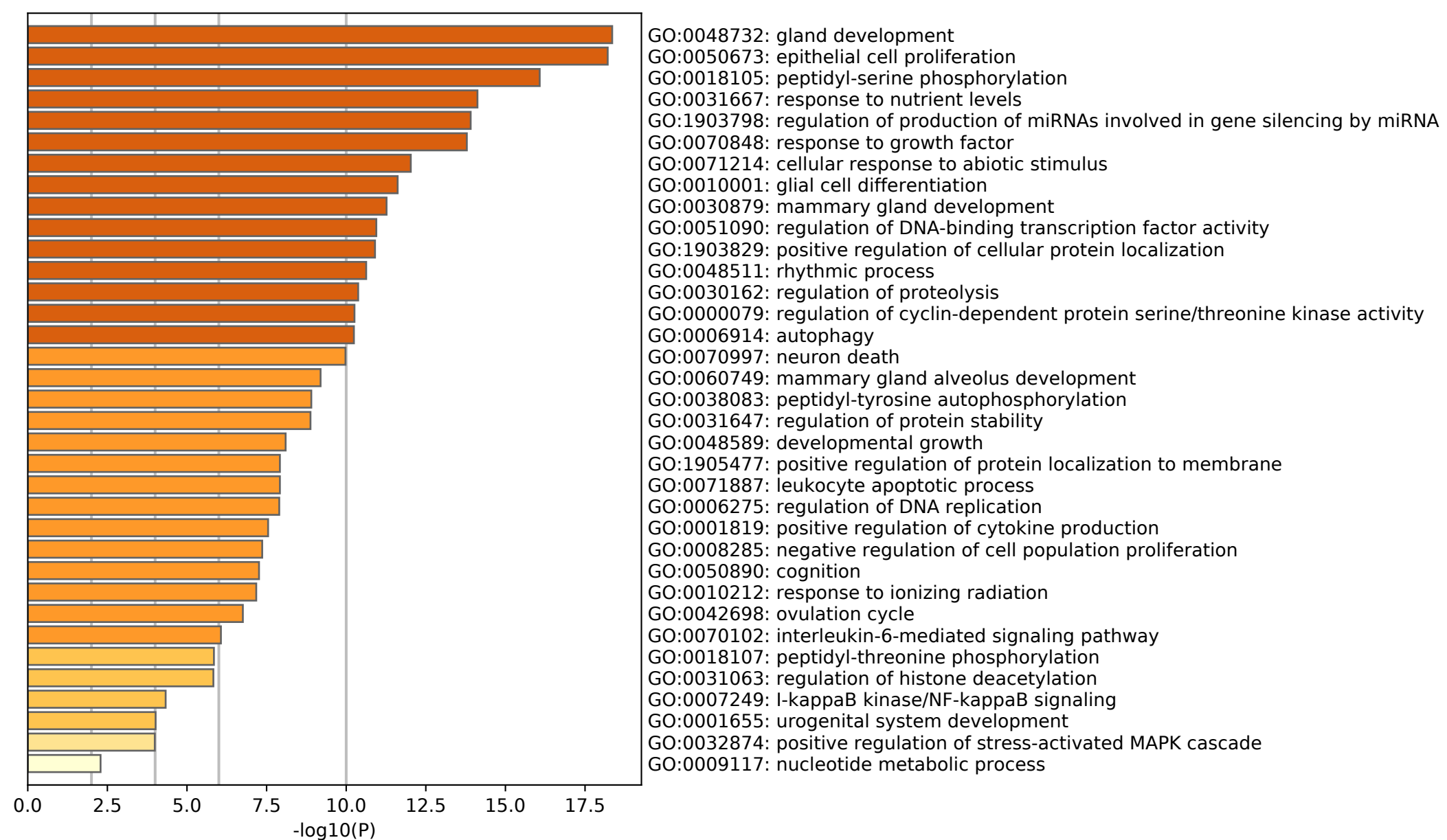

Supplement: Supplemental Information 3 [file peerj-10-13737-s003.zip › Enrichment_heatmap/HeatmapSelectedGOTop100.pdf]

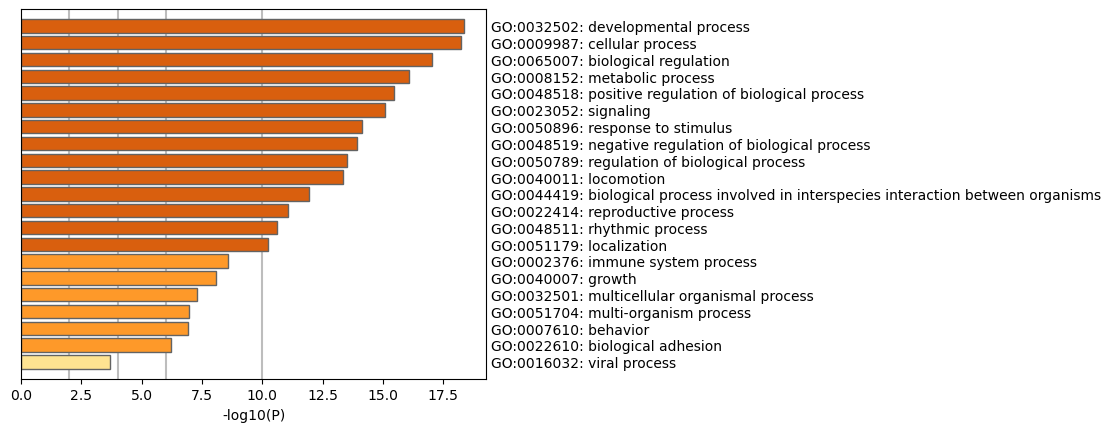

Supplement: Supplemental Information 3 [file peerj-10-13737-s003.zip › Enrichment_heatmap/HeatmapSelectedGOParent.png]

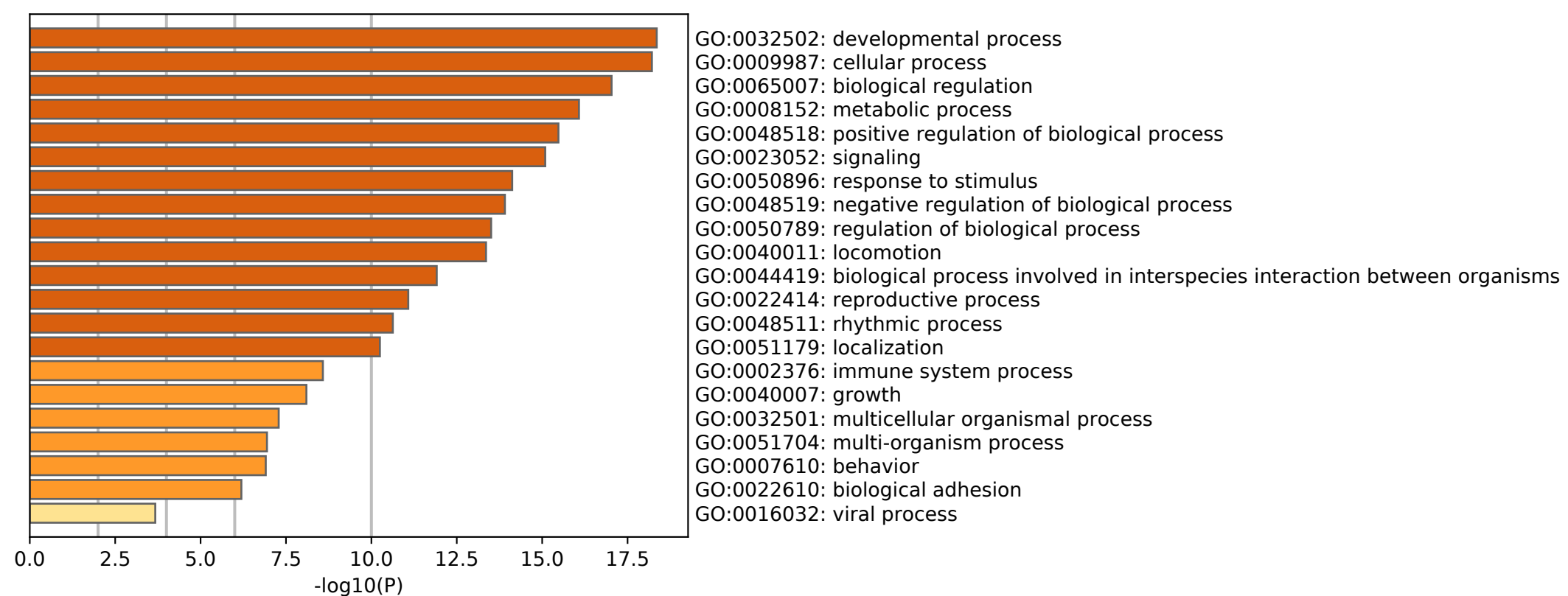

Supplement: Supplemental Information 3 [file peerj-10-13737-s003.zip › Enrichment_heatmap/HeatmapSelectedGOParent.pdf]

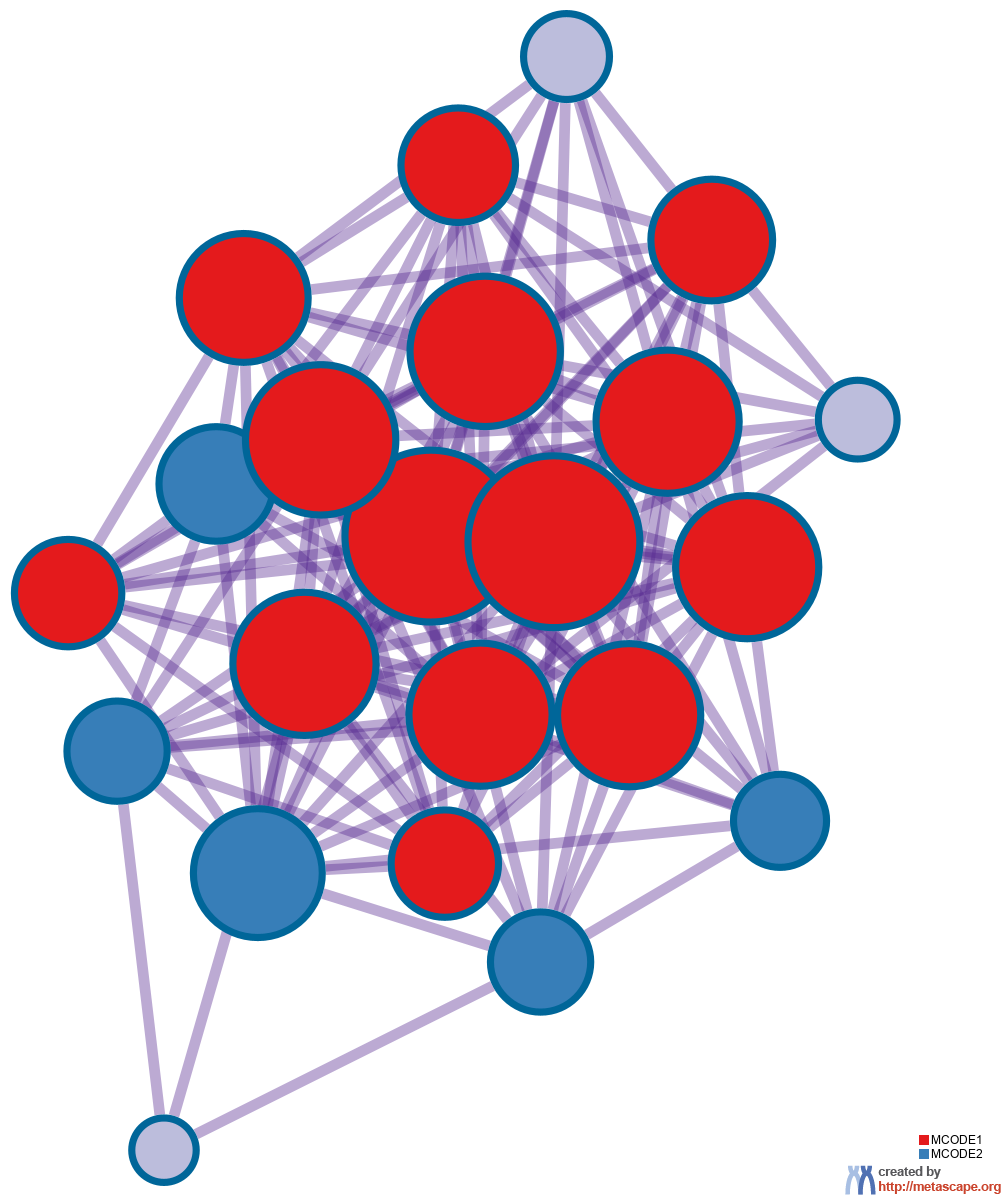

Supplement: Supplemental Information 3 [file peerj-10-13737-s003.zip › Enrichment_PPI/MyList_PPIColorByCluster.png]

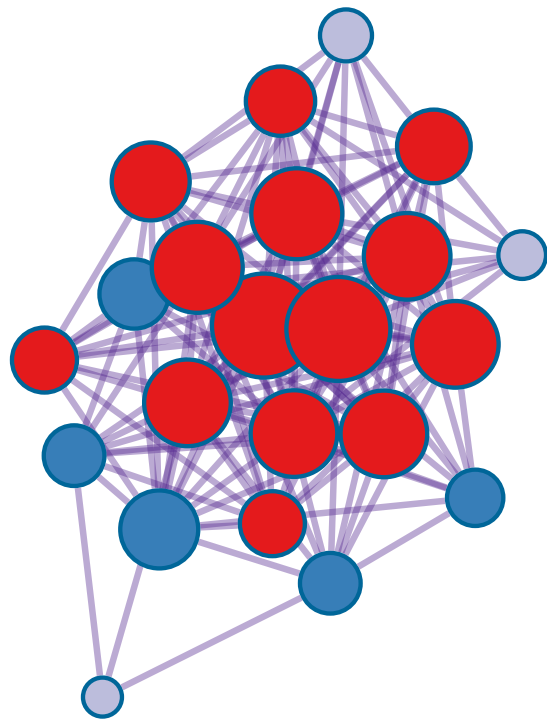

■ MCODE1  
■ MCODE2  
created by  
<http://metascape.org>

Supplement: Supplemental Information 3 [file peerj-10-13737-s003.zip › Enrichment_PPI/MyList_PPIColorByCluster.pdf]

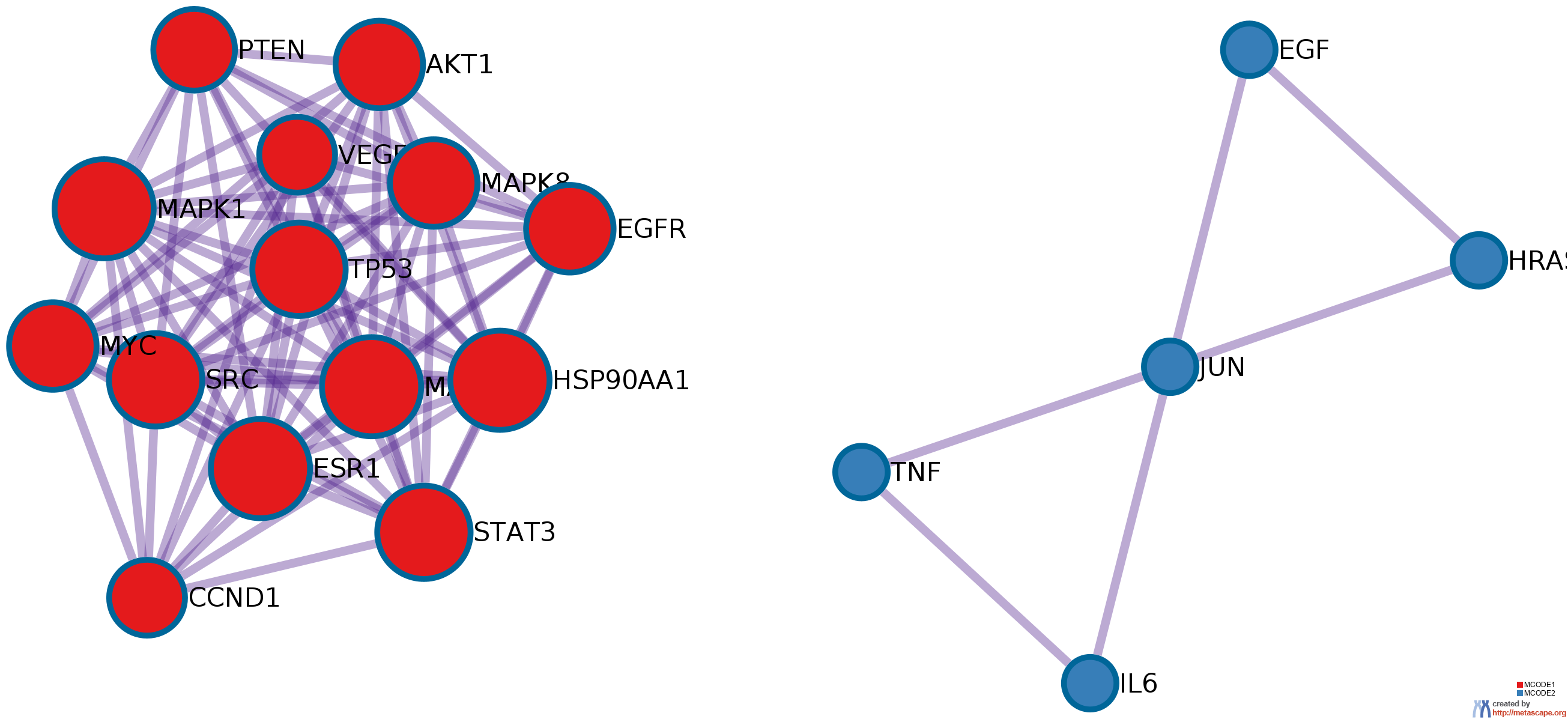

Supplement: Supplemental Information 3 [file peerj-10-13737-s003.zip › Enrichment_PPI/MyList_MCODE_ALL_PPIColorByCluster.png]

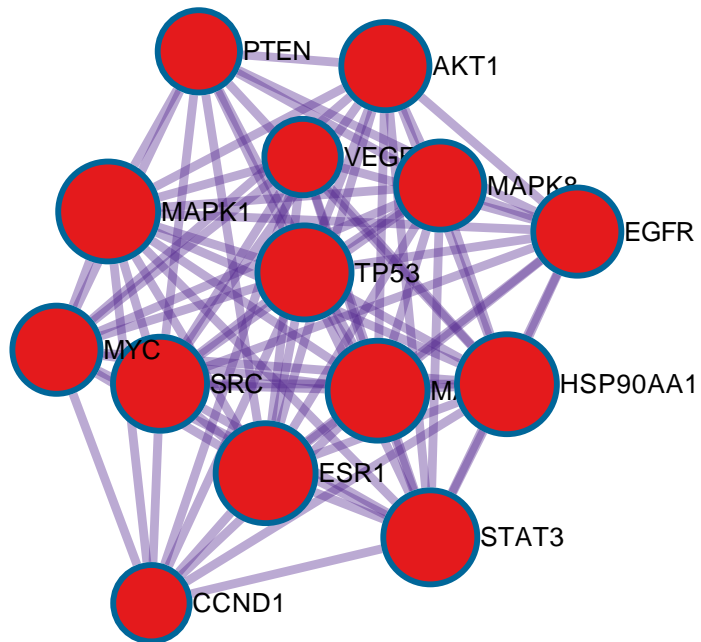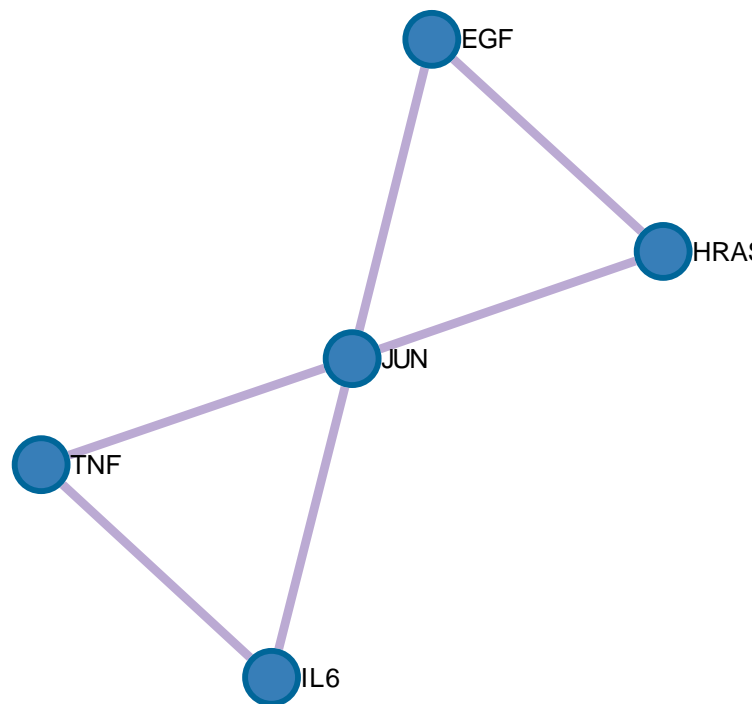

■ MCODE1  
■ MCODE2  
created by  
<http://metascape.org>

Supplement: Supplemental Information 3 [file peerj-10-13737-s003.zip › Enrichment_PPI/MyList_MCODE_ALL_PPIColorByCluster.pdf]

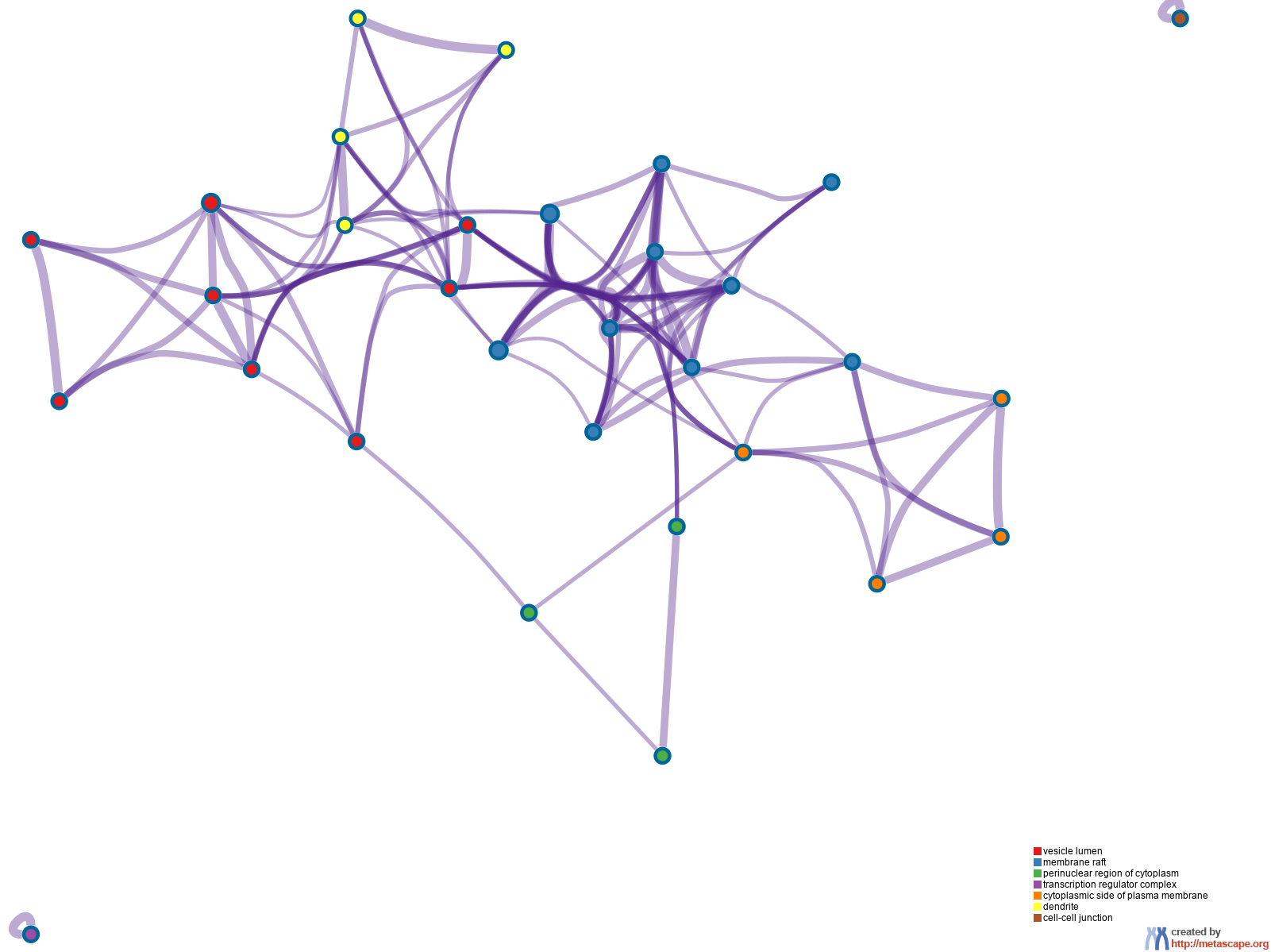

Supplement: Supplemental Information 4 [file peerj-10-13737-s004.zip › Enrichment_GO/ColorByCluster.png]

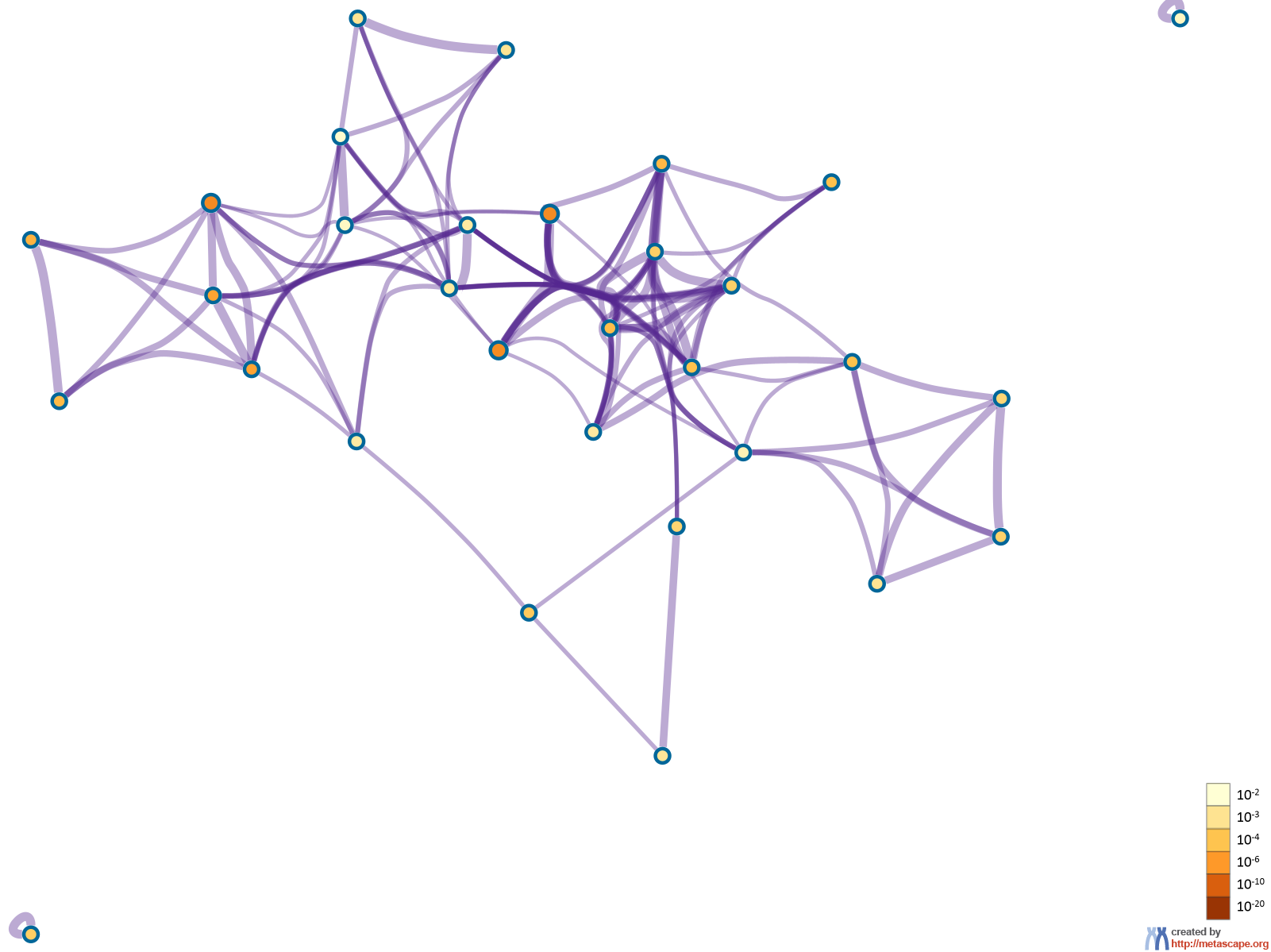

Supplement: Supplemental Information 4 [file peerj-10-13737-s004.zip › Enrichment_GO/ColorByPValue.png]

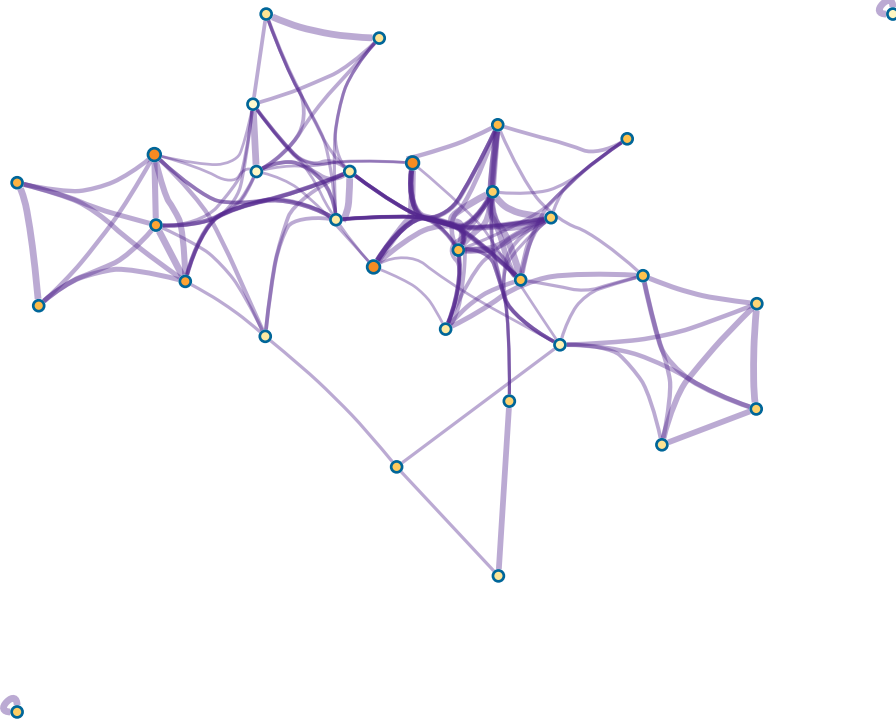

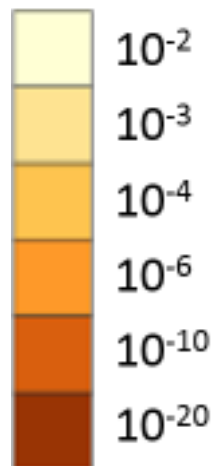

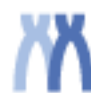 created by  
<http://metascape.org>

Supplement: Supplemental Information 4 [file peerj-10-13737-s004.zip › Enrichment_GO/ColorByPValue.pdf]

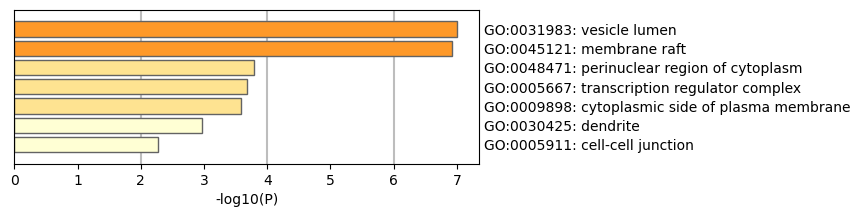

Supplement: Supplemental Information 4 [file peerj-10-13737-s004.zip › Enrichment_heatmap/HeatmapSelectedGO.png]

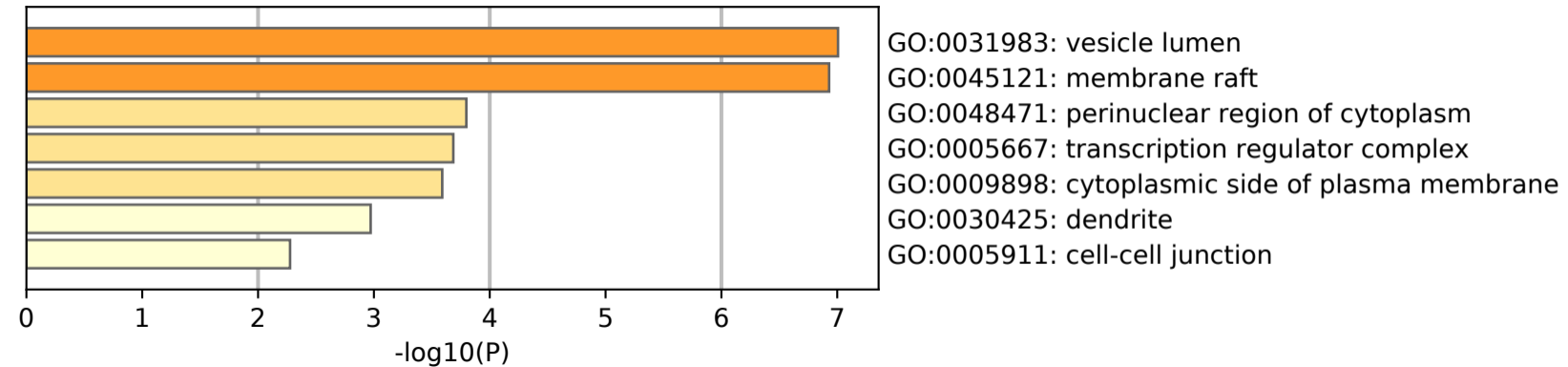

Supplement: Supplemental Information 4 [file peerj-10-13737-s004.zip › Enrichment_heatmap/HeatmapSelectedGO.pdf]

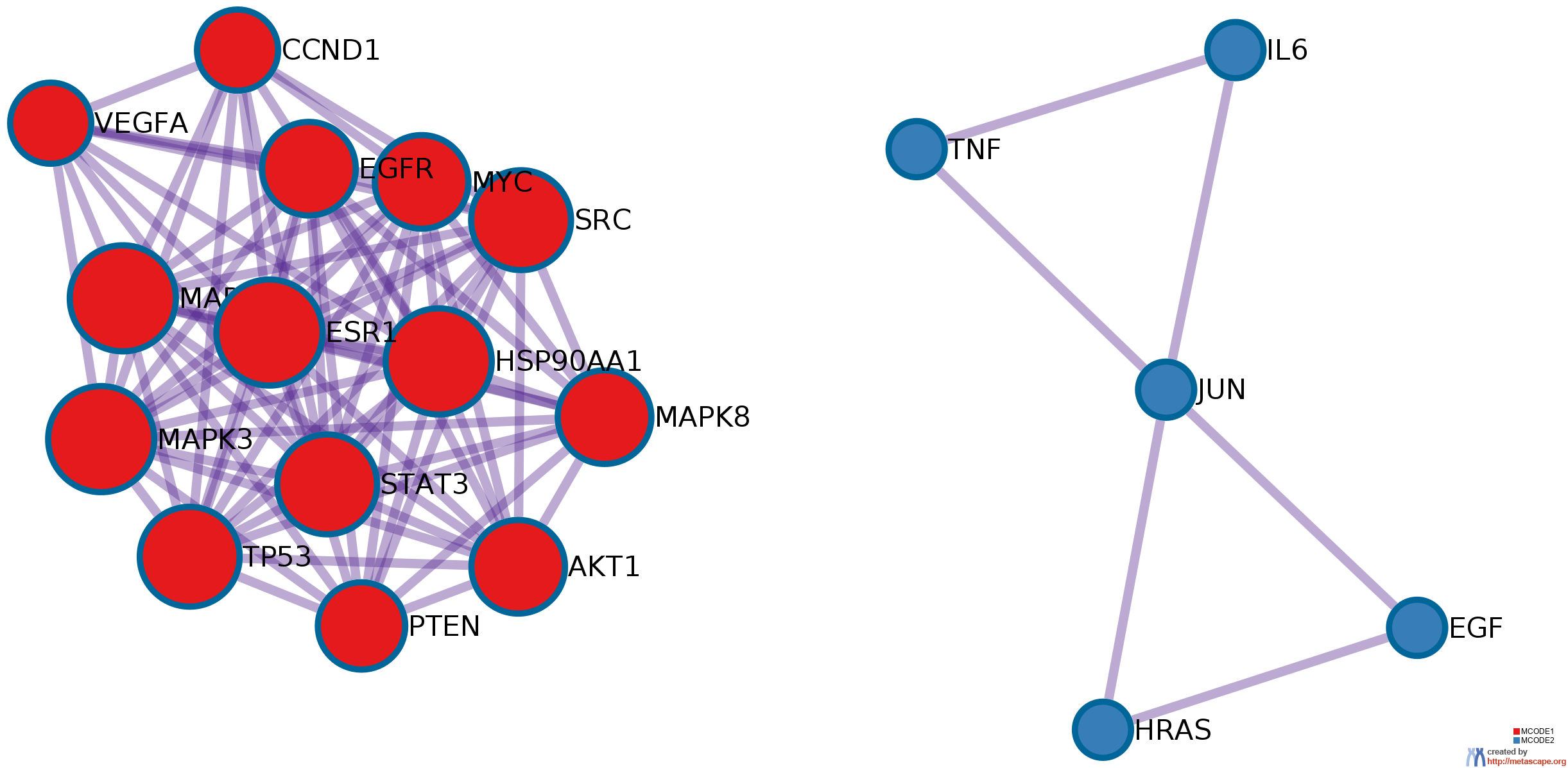

Supplement: Supplemental Information 4 [file peerj-10-13737-s004.zip › Enrichment_PPI/MyList_MCODE_ALL_PPIColorByCluster.png]

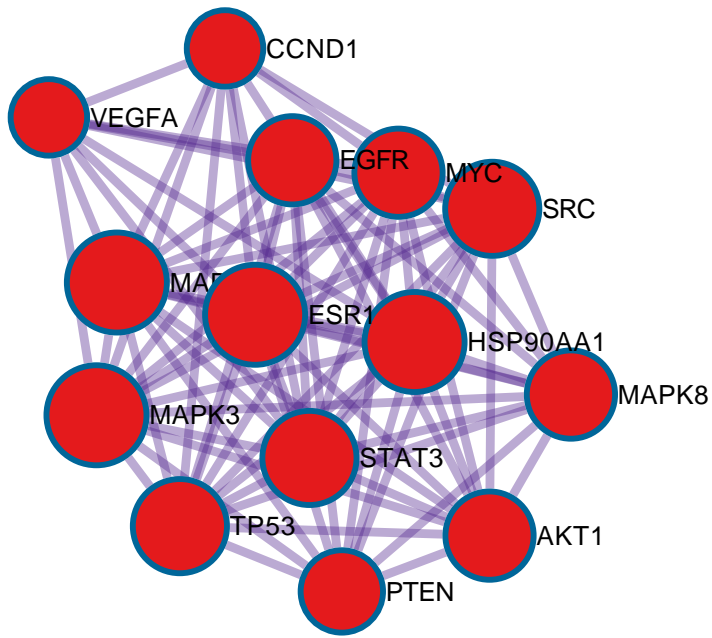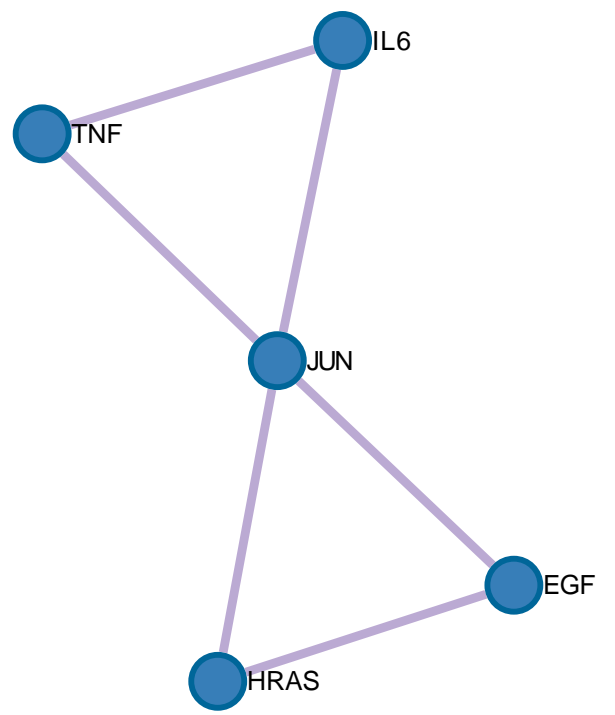

■ MCODE1  
■ MCODE2

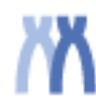 created by  
<http://metascape.org>

Supplement: Supplemental Information 4 [file peerj-10-13737-s004.zip › Enrichment_PPI/MyList_MCODE_ALL_PPIColorByCluster.pdf]

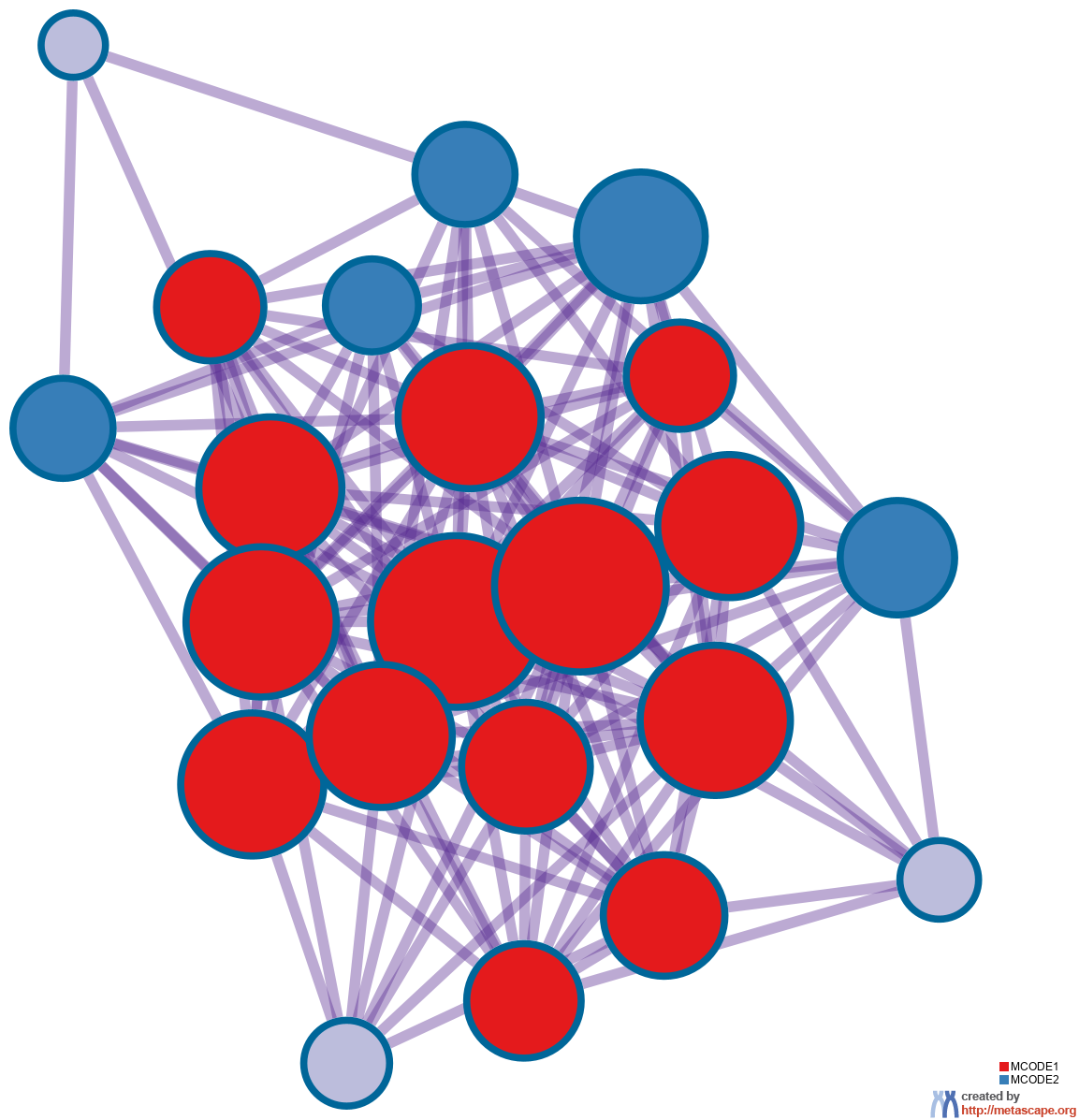

Supplement: Supplemental Information 4 [file peerj-10-13737-s004.zip › Enrichment_PPI/MyList_PPIColorByCluster.png]

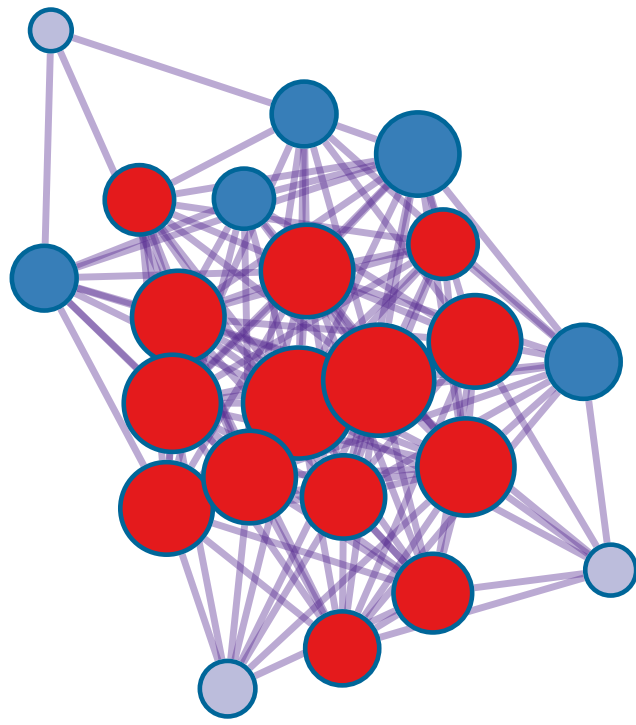

■ MCODE1  
■ MCODE2

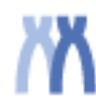 created by  
<http://metascape.org>

Supplement: Supplemental Information 4 [file peerj-10-13737-s004.zip › Enrichment_PPI/MyList_PPIColorByCluster.pdf]

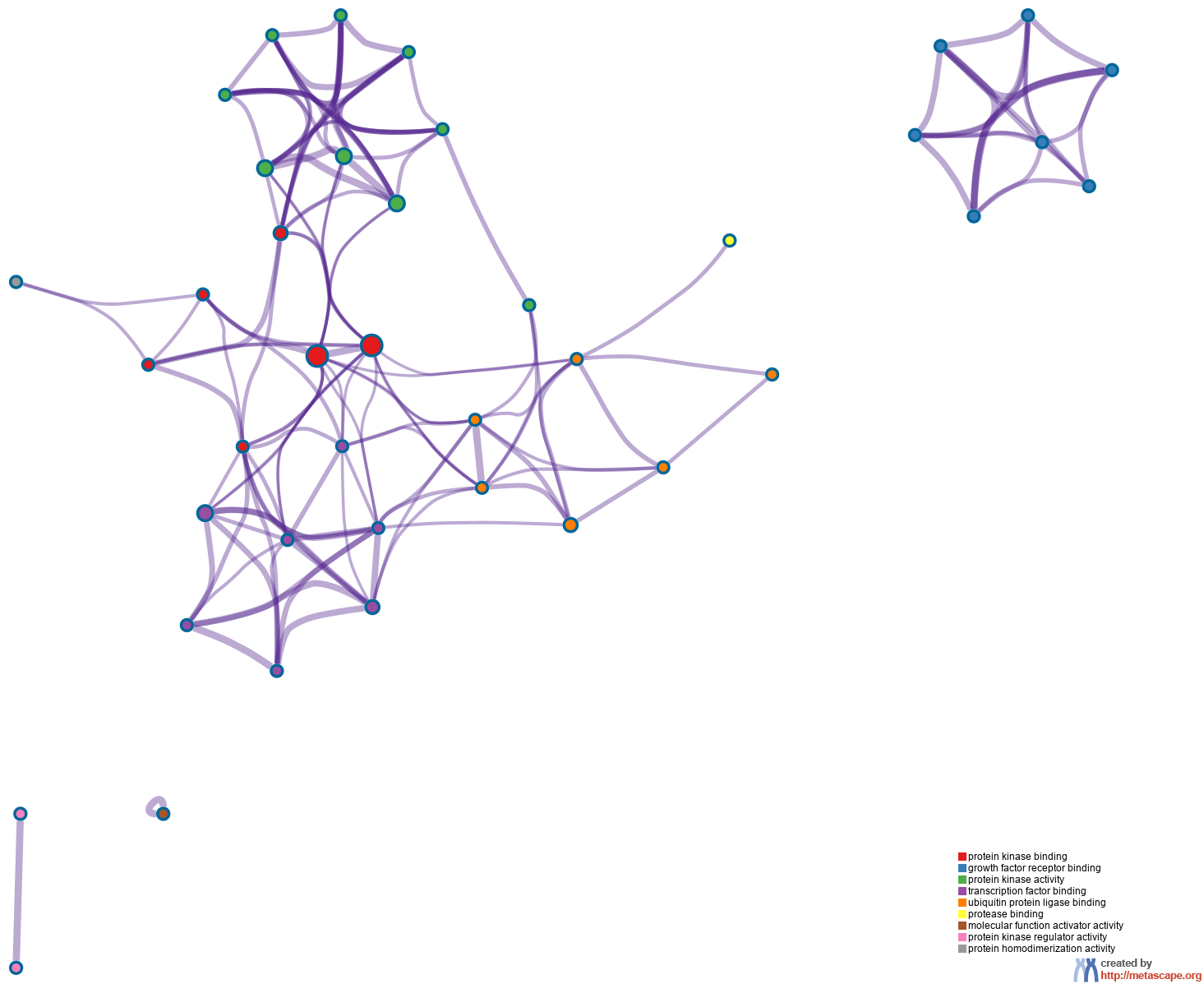

Supplement: Supplemental Information 5 [file peerj-10-13737-s005.zip › Enrichment_GO/ColorByCluster.png]

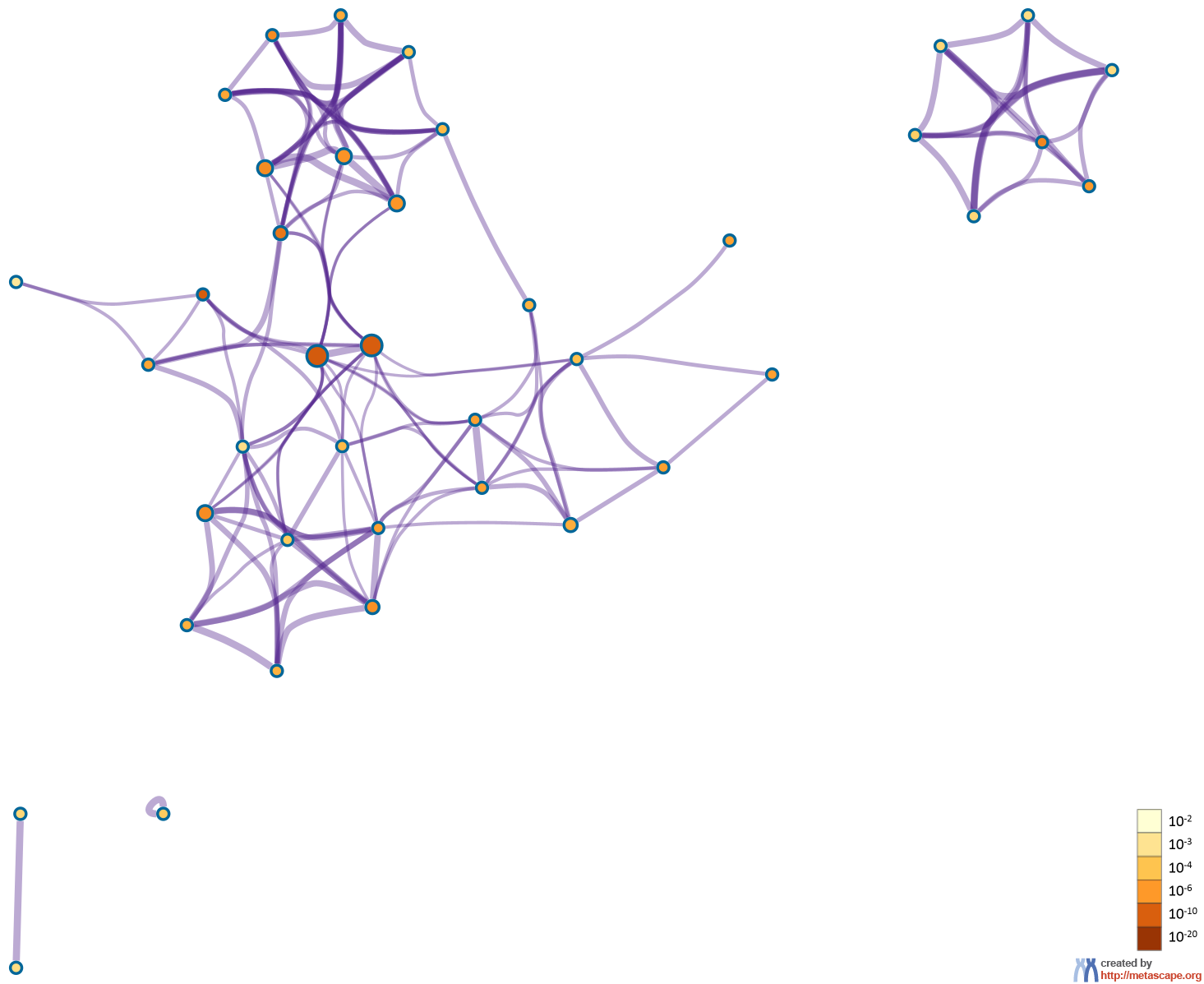

Supplement: Supplemental Information 5 [file peerj-10-13737-s005.zip › Enrichment_GO/ColorByPValue.png]

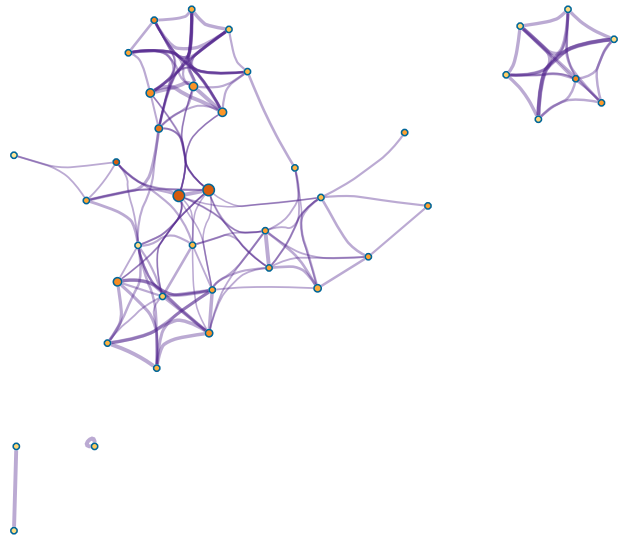

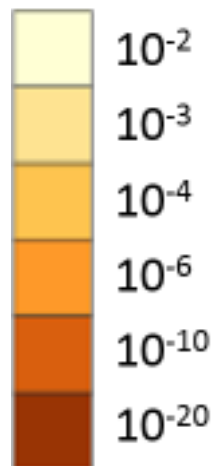

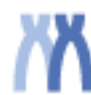 created by  
<http://metascape.org>

Supplement: Supplemental Information 5 [file peerj-10-13737-s005.zip › Enrichment_GO/ColorByPValue.pdf]

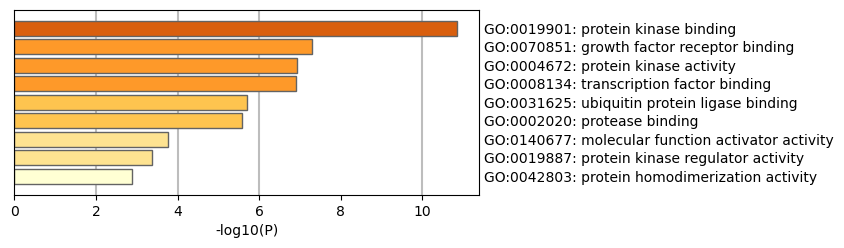

Supplement: Supplemental Information 5 [file peerj-10-13737-s005.zip › Enrichment_heatmap/HeatmapSelectedGO.png]

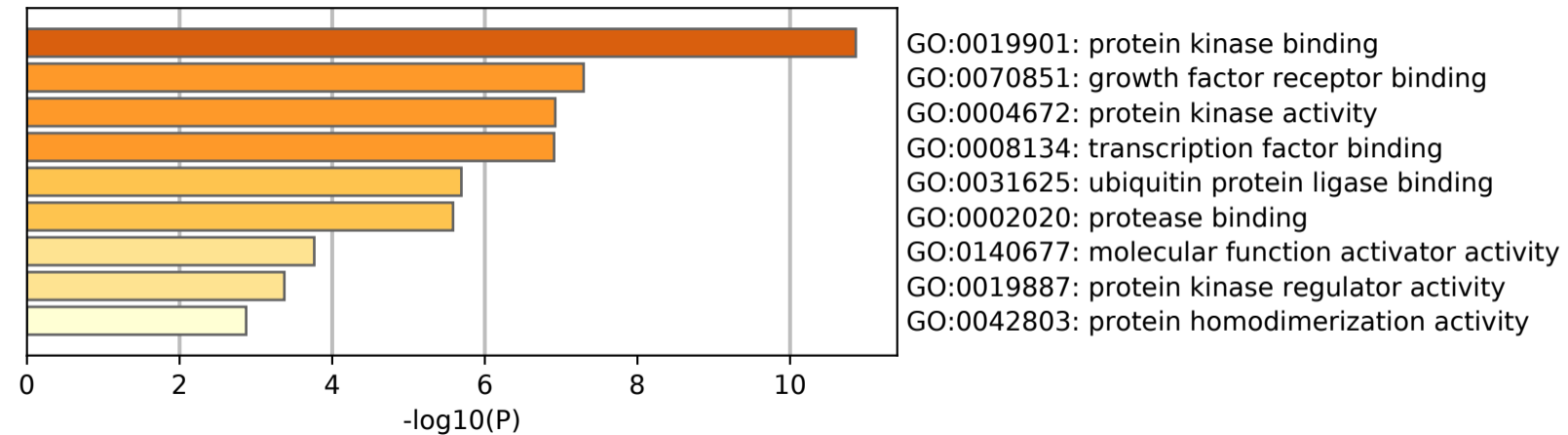

Supplement: Supplemental Information 5 [file peerj-10-13737-s005.zip › Enrichment_heatmap/HeatmapSelectedGO.pdf]

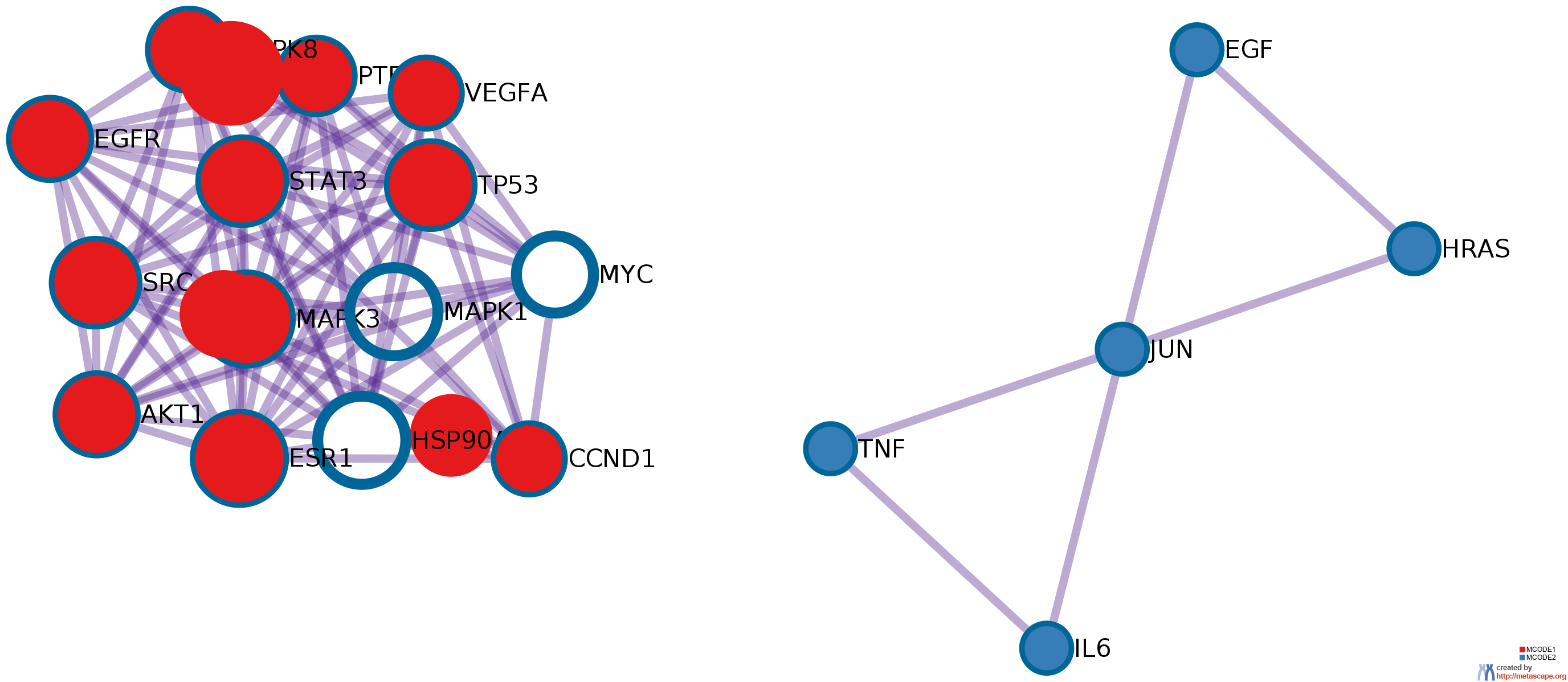

Supplement: Supplemental Information 5 [file peerj-10-13737-s005.zip › Enrichment_PPI/MyList_MCODE_ALL_PPIColorByCluster.png]

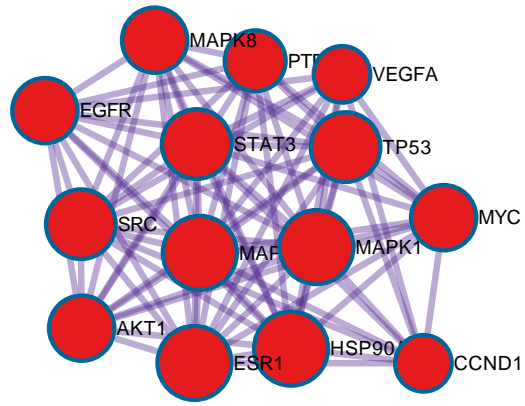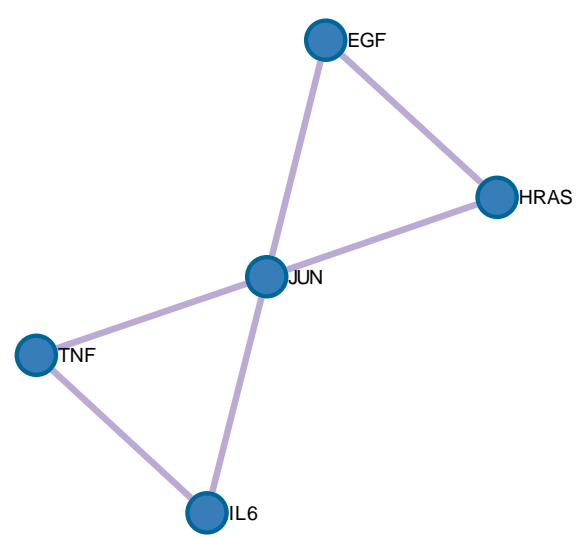

■ MCODE1  
■ MCODE2  
created by  
<http://metascape.org>

Supplement: Supplemental Information 5 [file peerj-10-13737-s005.zip › Enrichment_PPI/MyList_MCODE_ALL_PPIColorByCluster.pdf]

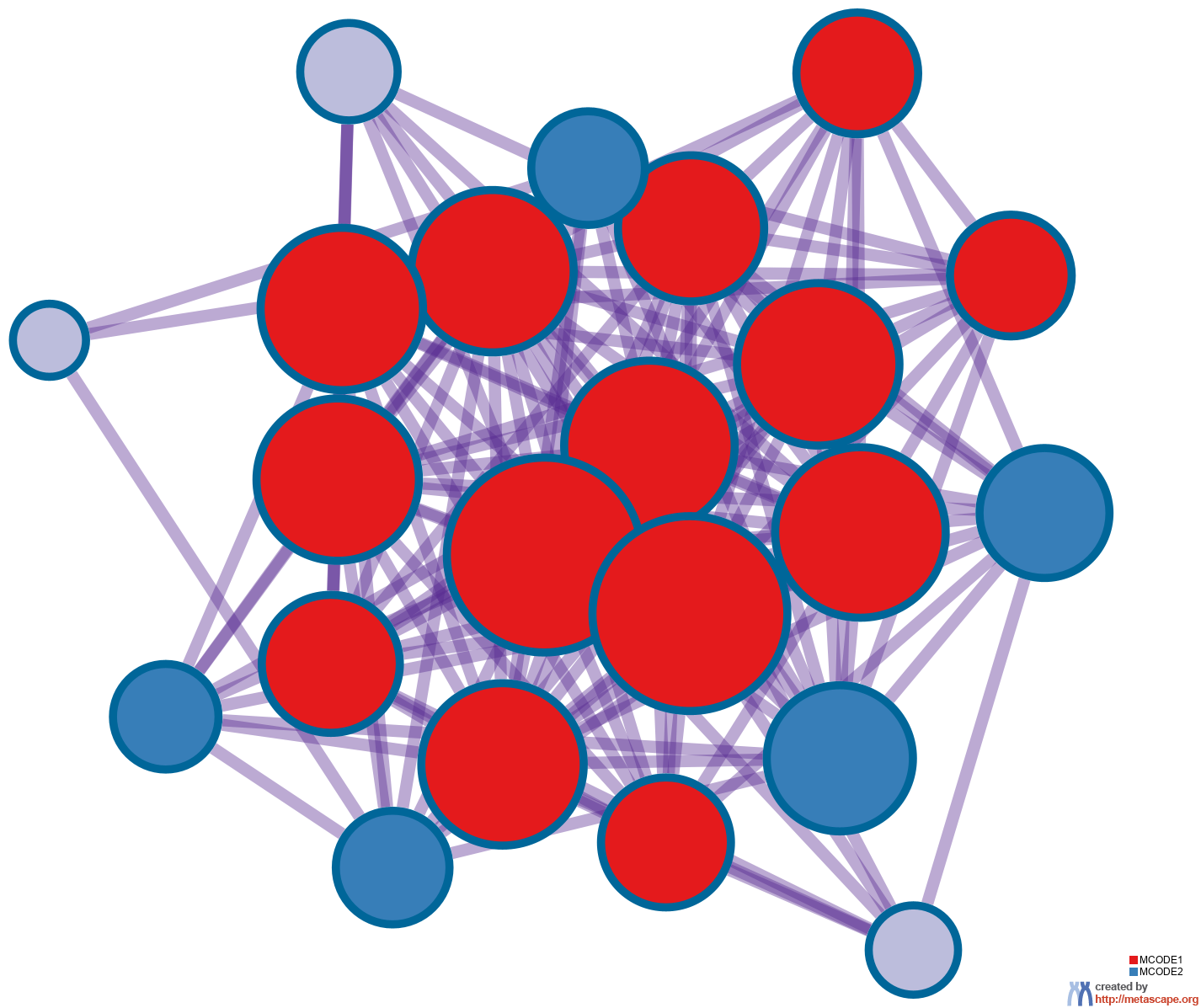

Supplement: Supplemental Information 5 [file peerj-10-13737-s005.zip › Enrichment_PPI/MyList_PPIColorByCluster.png]

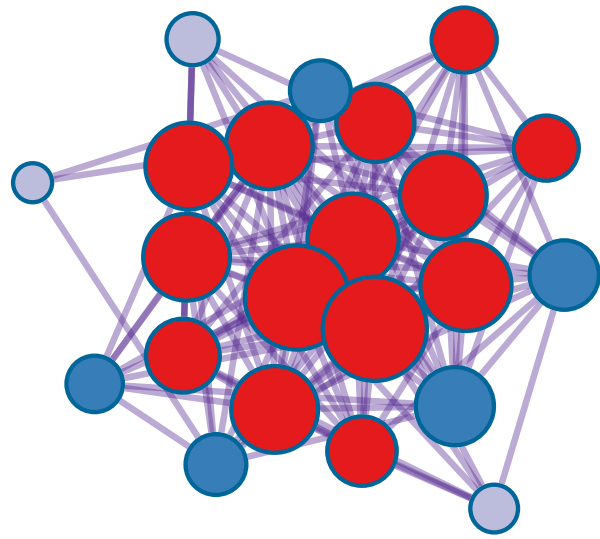

■ MCODE1  
■ MCODE2  
created by  
<http://metascape.org>

Supplement: Supplemental Information 5 [file peerj-10-13737-s005.zip › Enrichment_PPI/MyList_PPIColorByCluster.pdf]

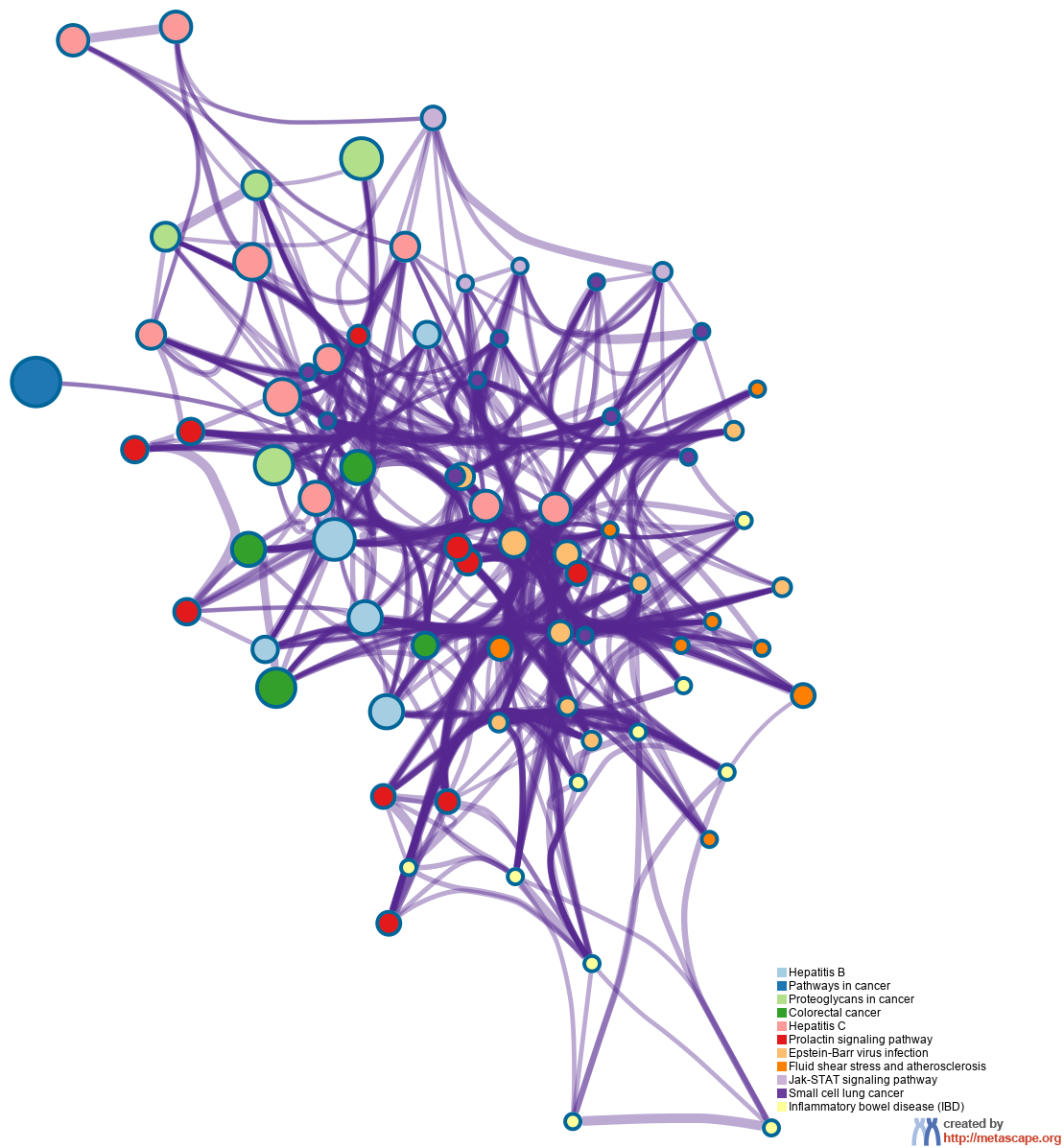

Supplement: Supplemental Information 6 [file peerj-10-13737-s006.zip › Enrichment_GO/ColorByCluster.png]

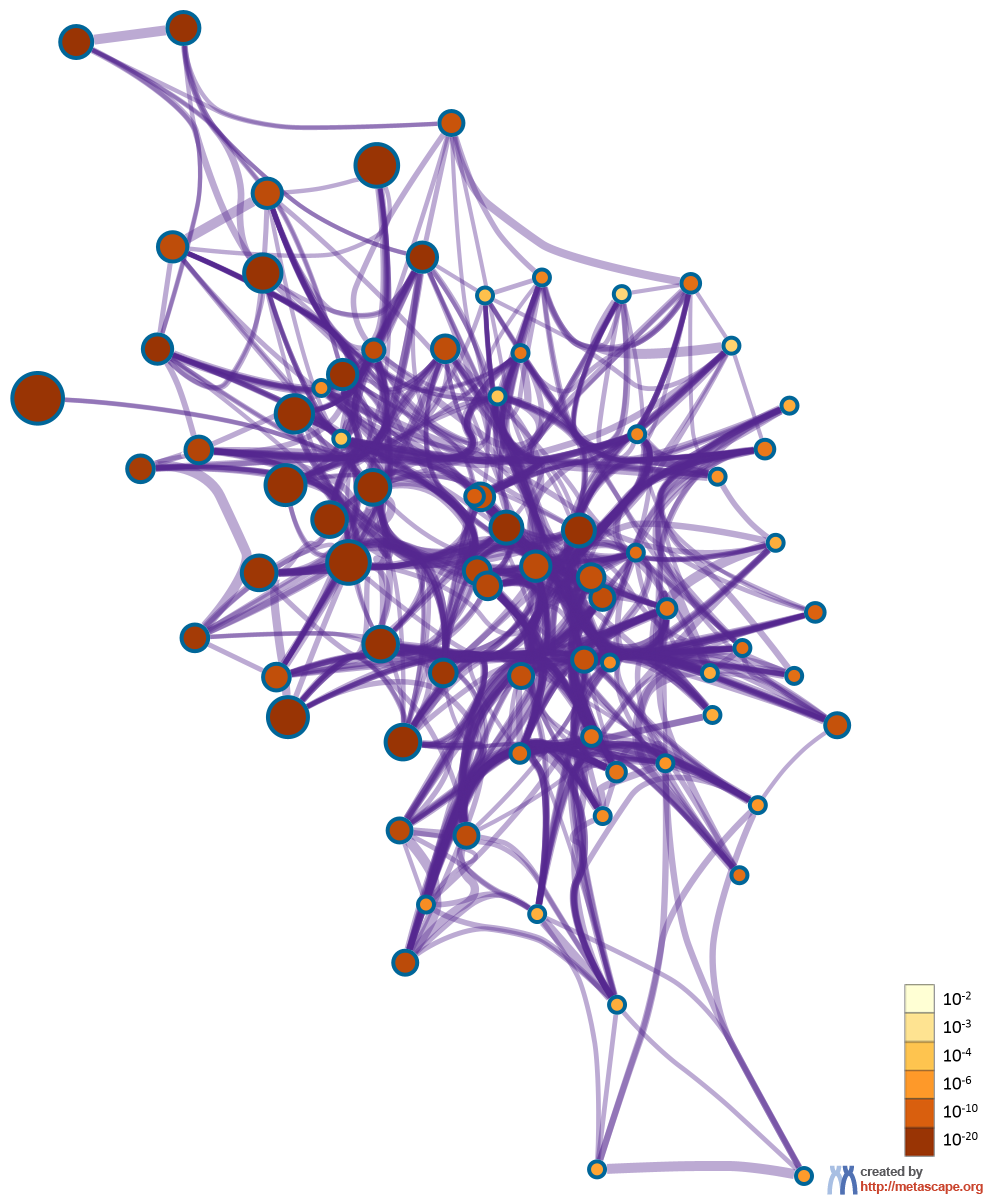

Supplement: Supplemental Information 6 [file peerj-10-13737-s006.zip › Enrichment_GO/ColorByPValue.png]

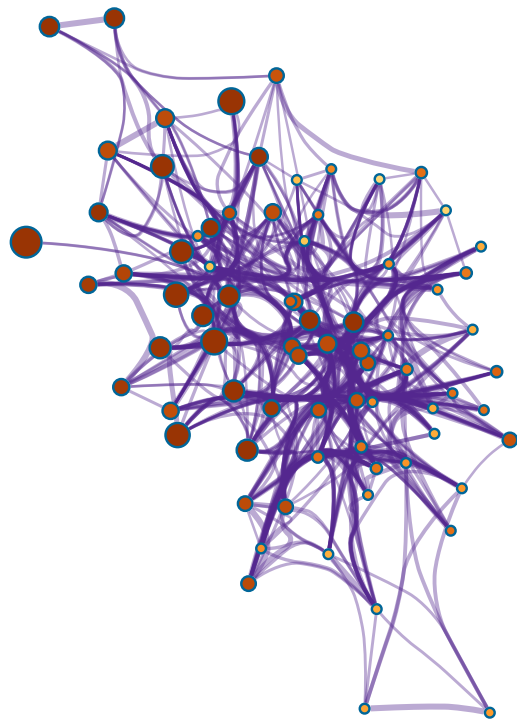

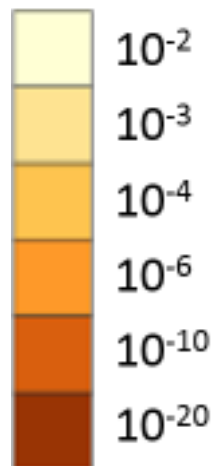

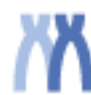 created by  
<http://metascape.org>

Supplement: Supplemental Information 6 [file peerj-10-13737-s006.zip › Enrichment_GO/ColorByPValue.pdf]

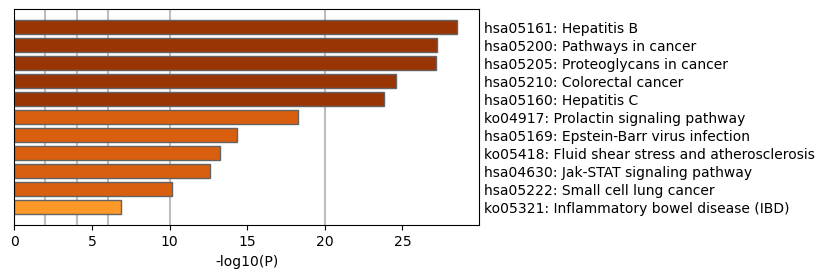

Supplement: Supplemental Information 6 [file peerj-10-13737-s006.zip › Enrichment_heatmap/HeatmapSelectedGO.png]

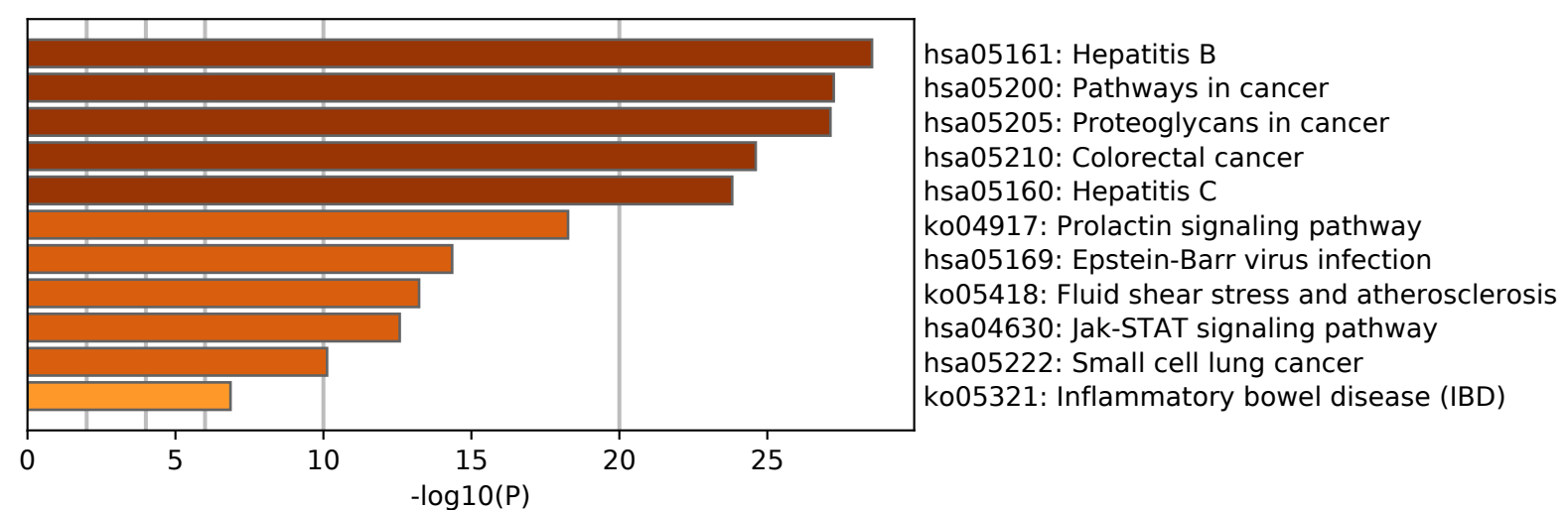

Supplement: Supplemental Information 6 [file peerj-10-13737-s006.zip › Enrichment_heatmap/HeatmapSelectedGO.pdf]

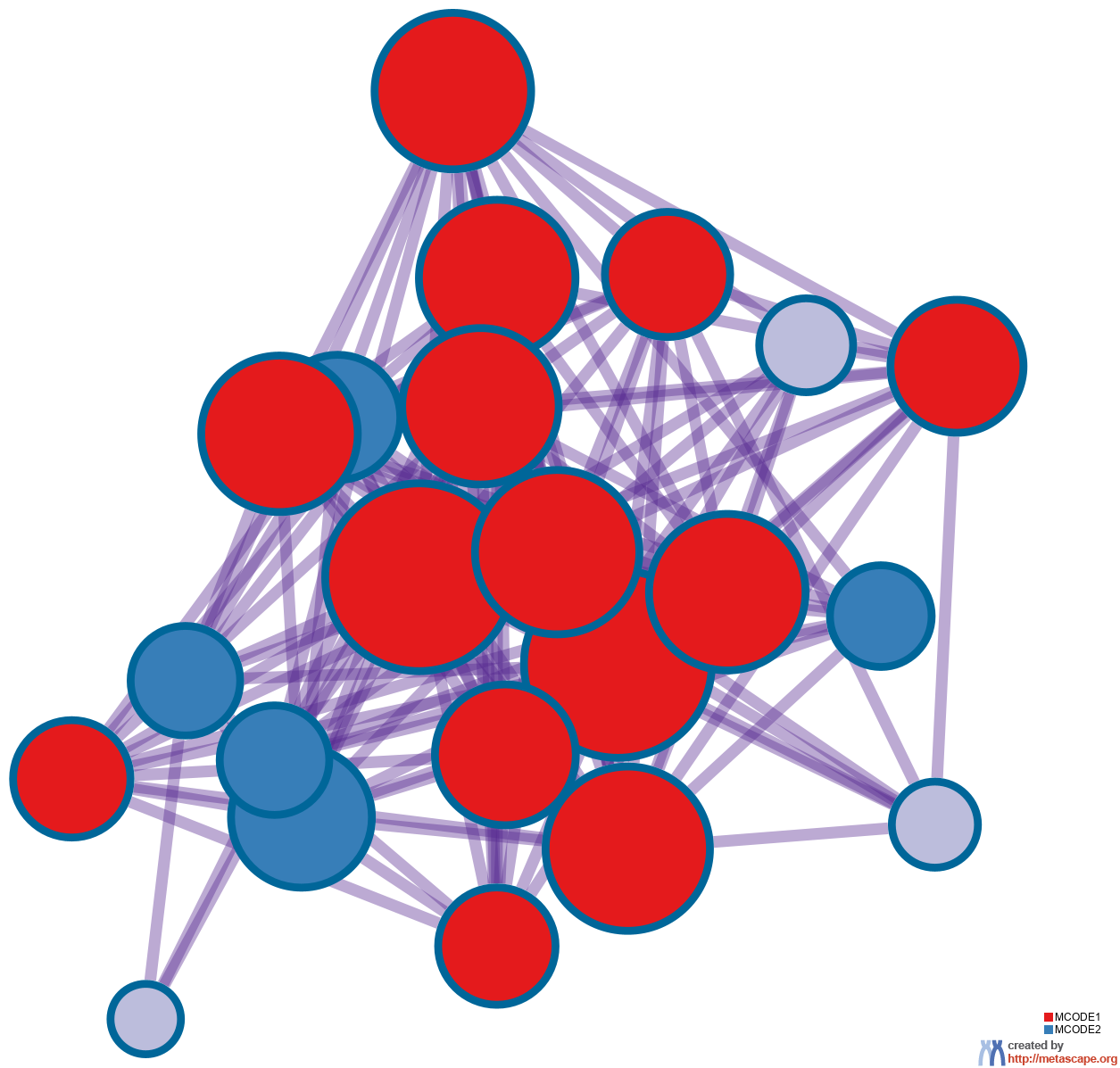

Supplement: Supplemental Information 6 [file peerj-10-13737-s006.zip › Enrichment_PPI/MyList_PPIColorByCluster.png]

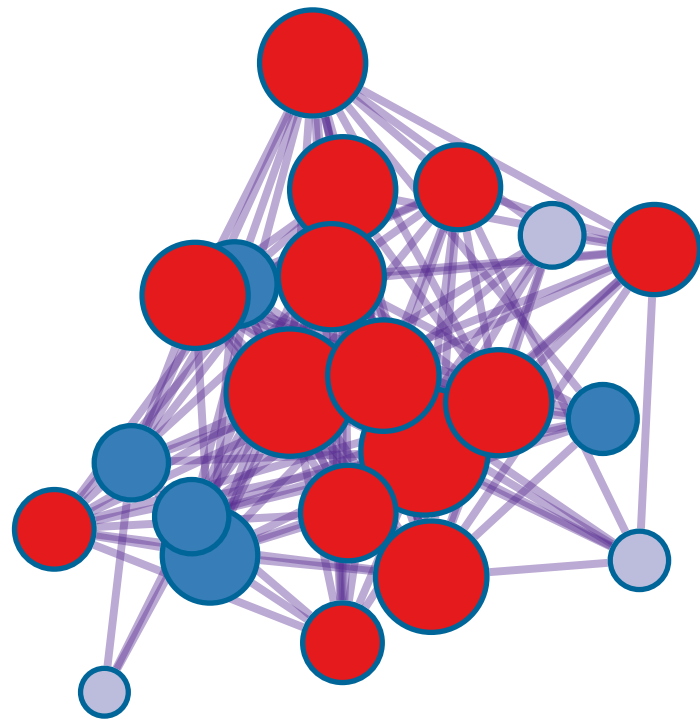

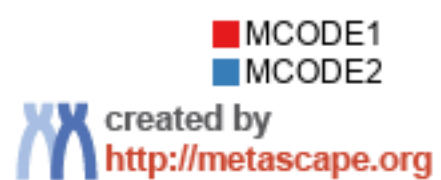

■ MCODE1  
■ MCODE2

Supplement: Supplemental Information 6 [file peerj-10-13737-s006.zip › Enrichment_PPI/MyList_PPIColorByCluster.pdf]

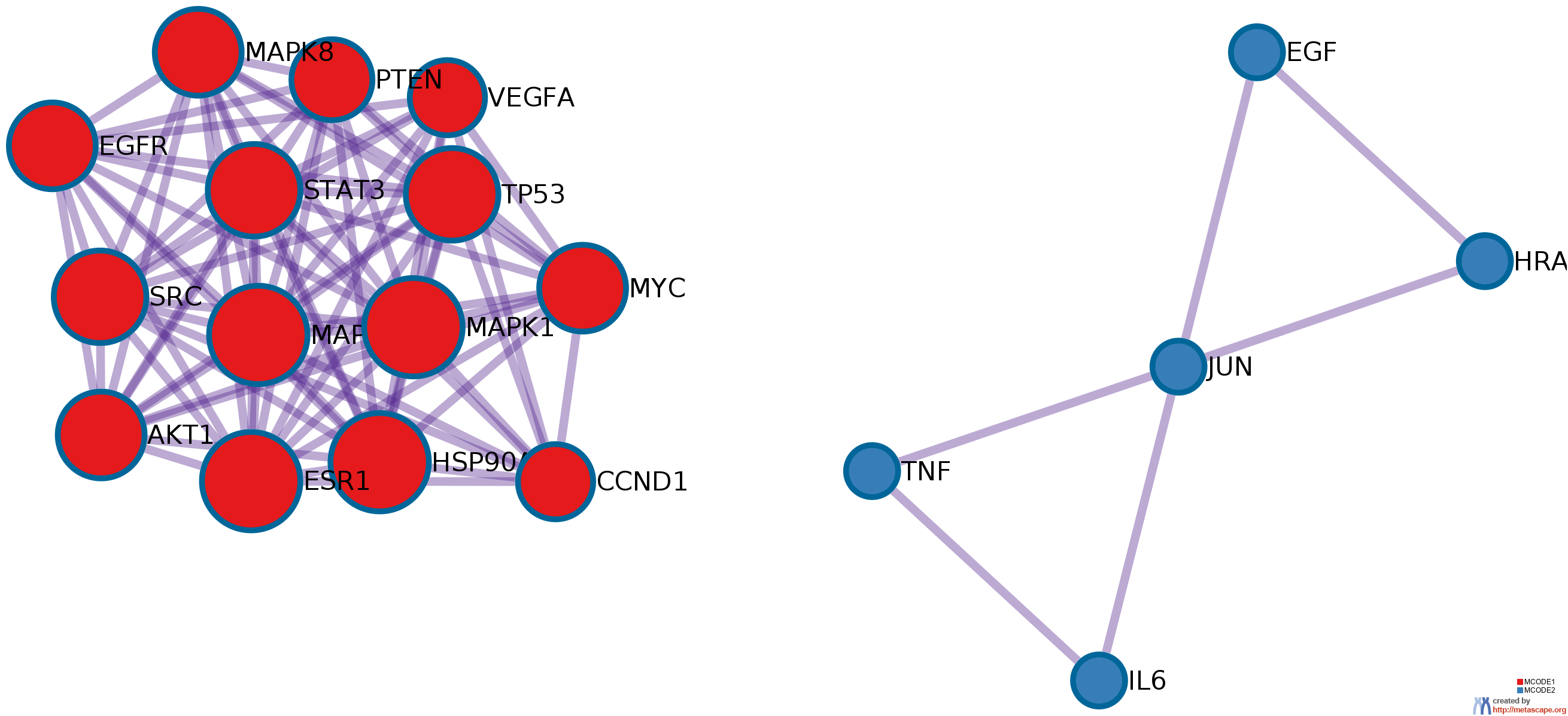

Supplement: Supplemental Information 6 [file peerj-10-13737-s006.zip › Enrichment_PPI/MyList_MCODE_ALL_PPIColorByCluster.png]

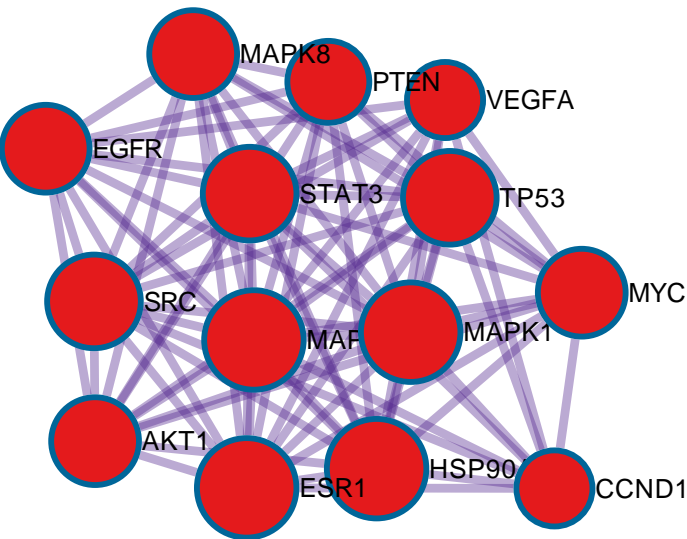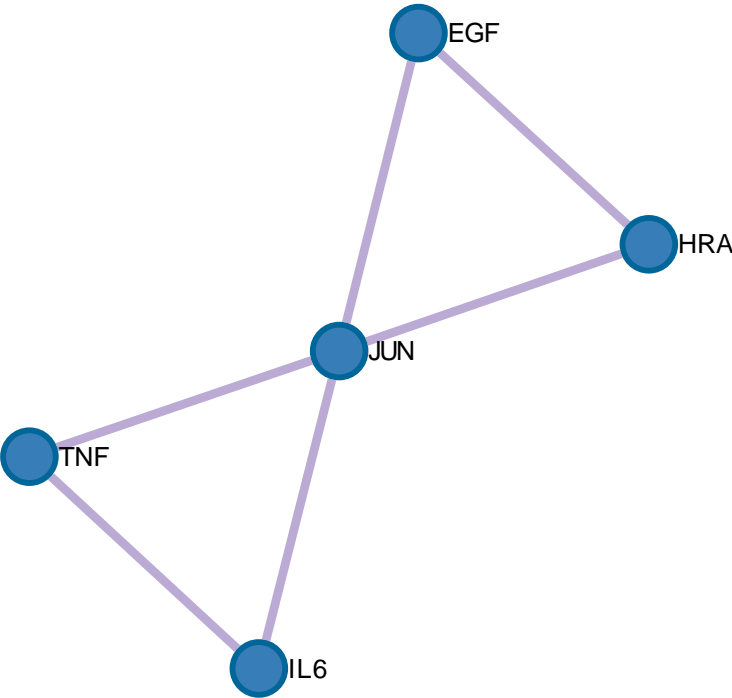

■ MCODE1  
■ MCODE2

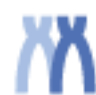 created by  
<http://metascape.org>

Supplement: Supplemental Information 6 [file peerj-10-13737-s006.zip › Enrichment_PPI/MyList_MCODE_ALL_PPIColorByCluster.pdf]

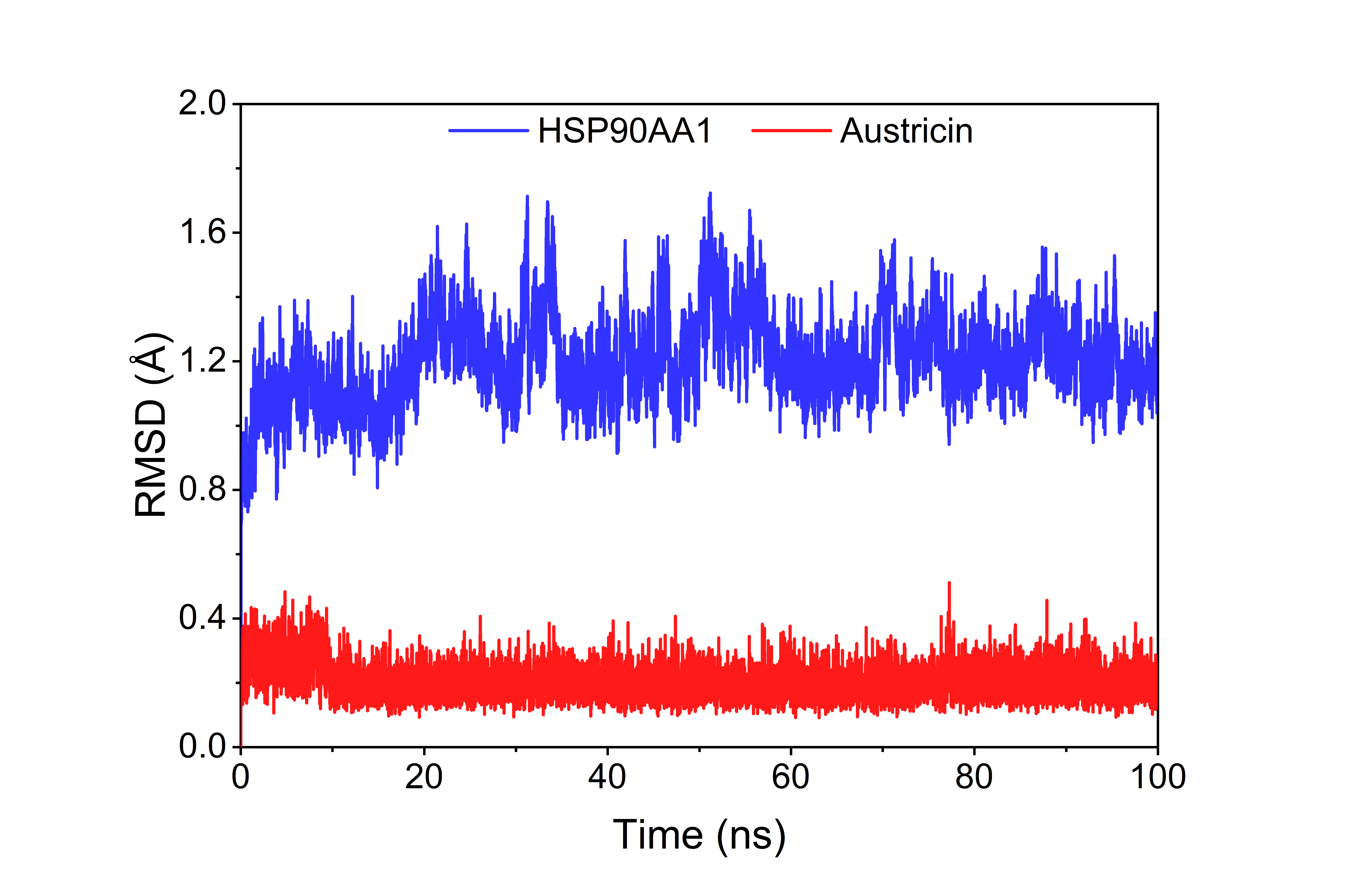

Supplement: Supplemental Information 7 [file peerj-10-13737-s007.zip › Molecular dynamics (MD) simulation data/Austricin-HSP90AA1/rmsd_HSP90AA1-austricin.tif]

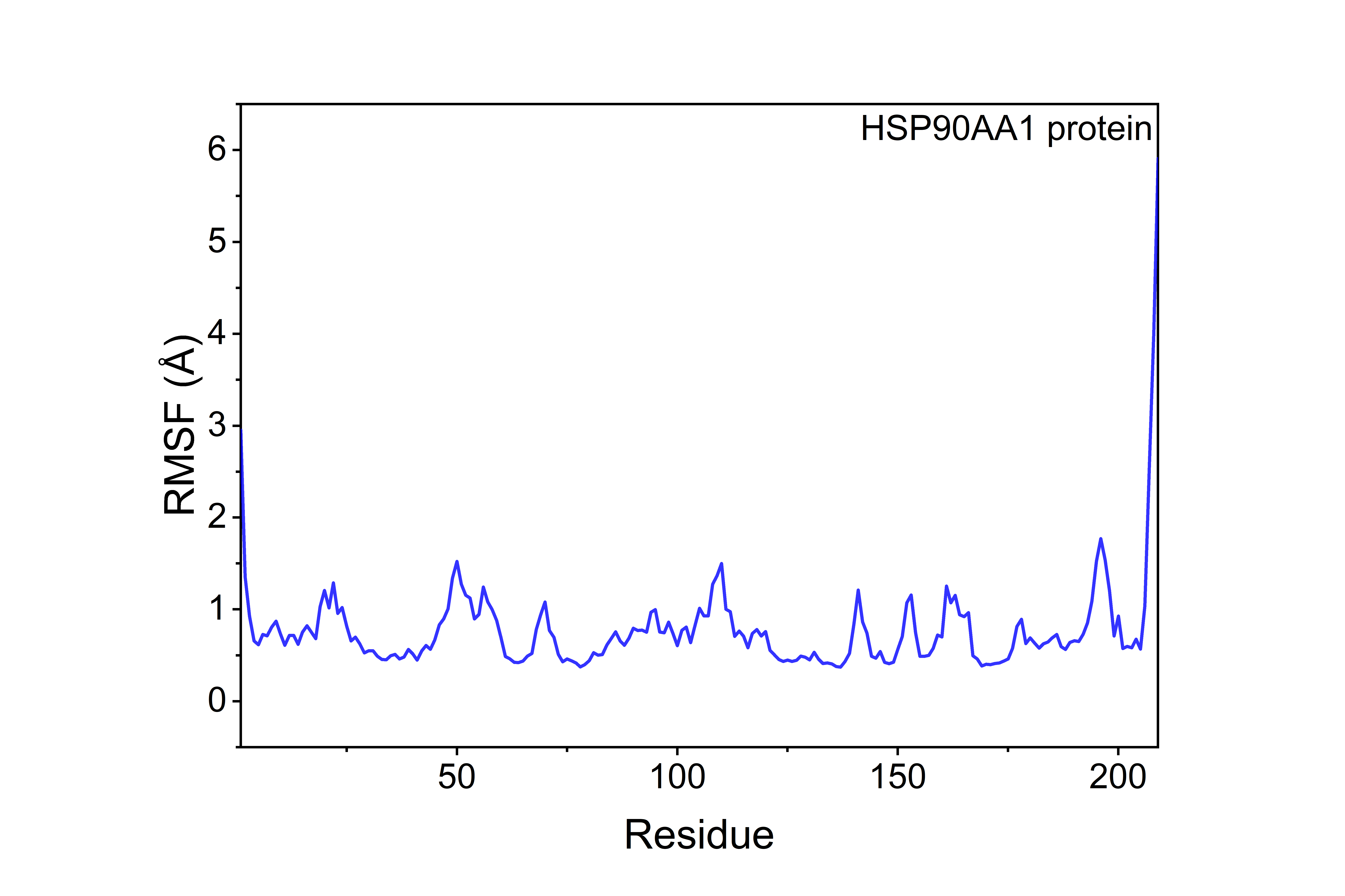

Supplement: Supplemental Information 7 [file peerj-10-13737-s007.zip › Molecular dynamics (MD) simulation data/Austricin-HSP90AA1/rmsf-HSP90AA1-austricin.tif]

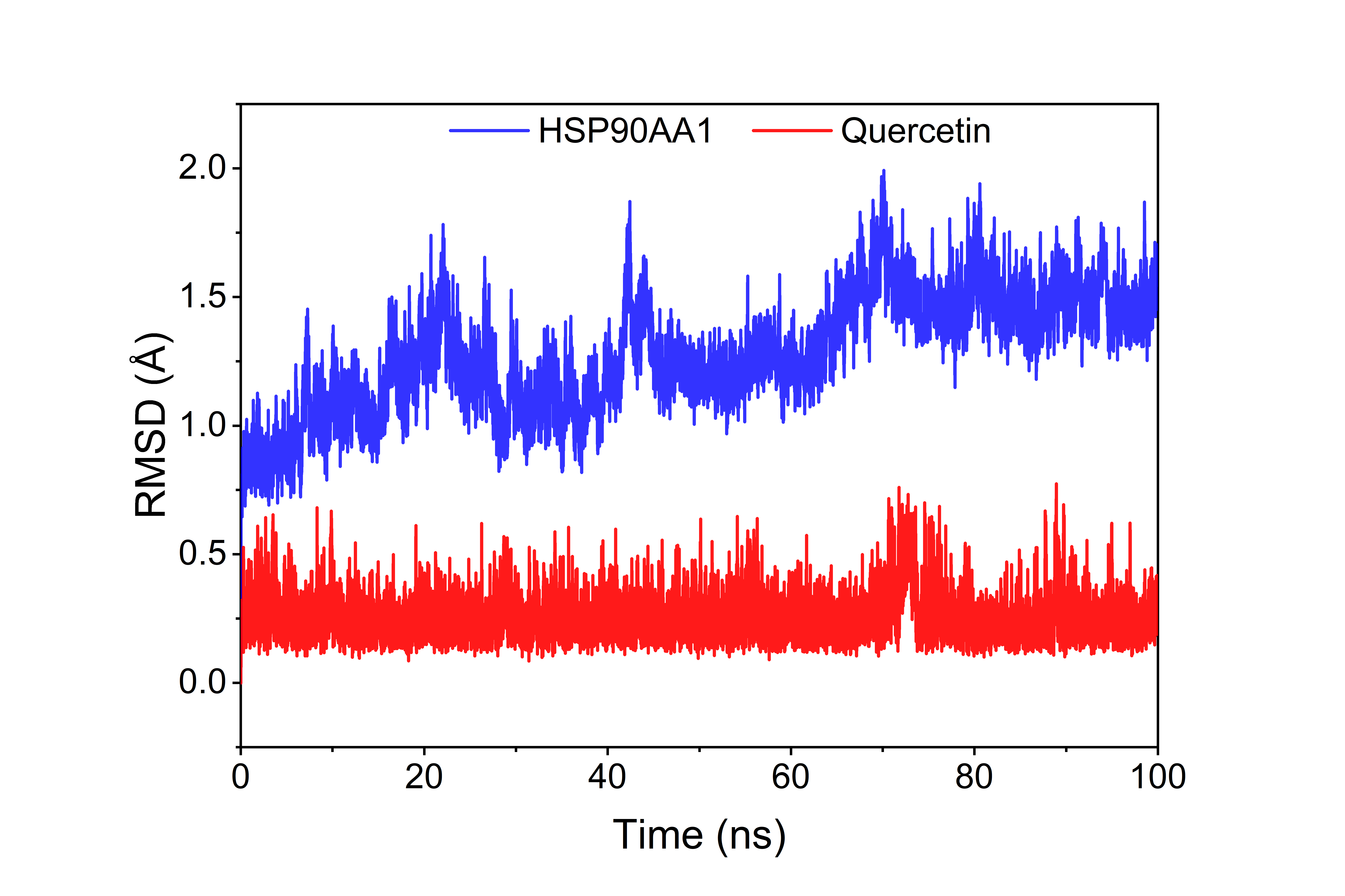

Supplement: Supplemental Information 7 [file peerj-10-13737-s007.zip › Molecular dynamics (MD) simulation data/HSP90AA1-Quercetin/rmsd_HSP90AA1-quercetin.tif]

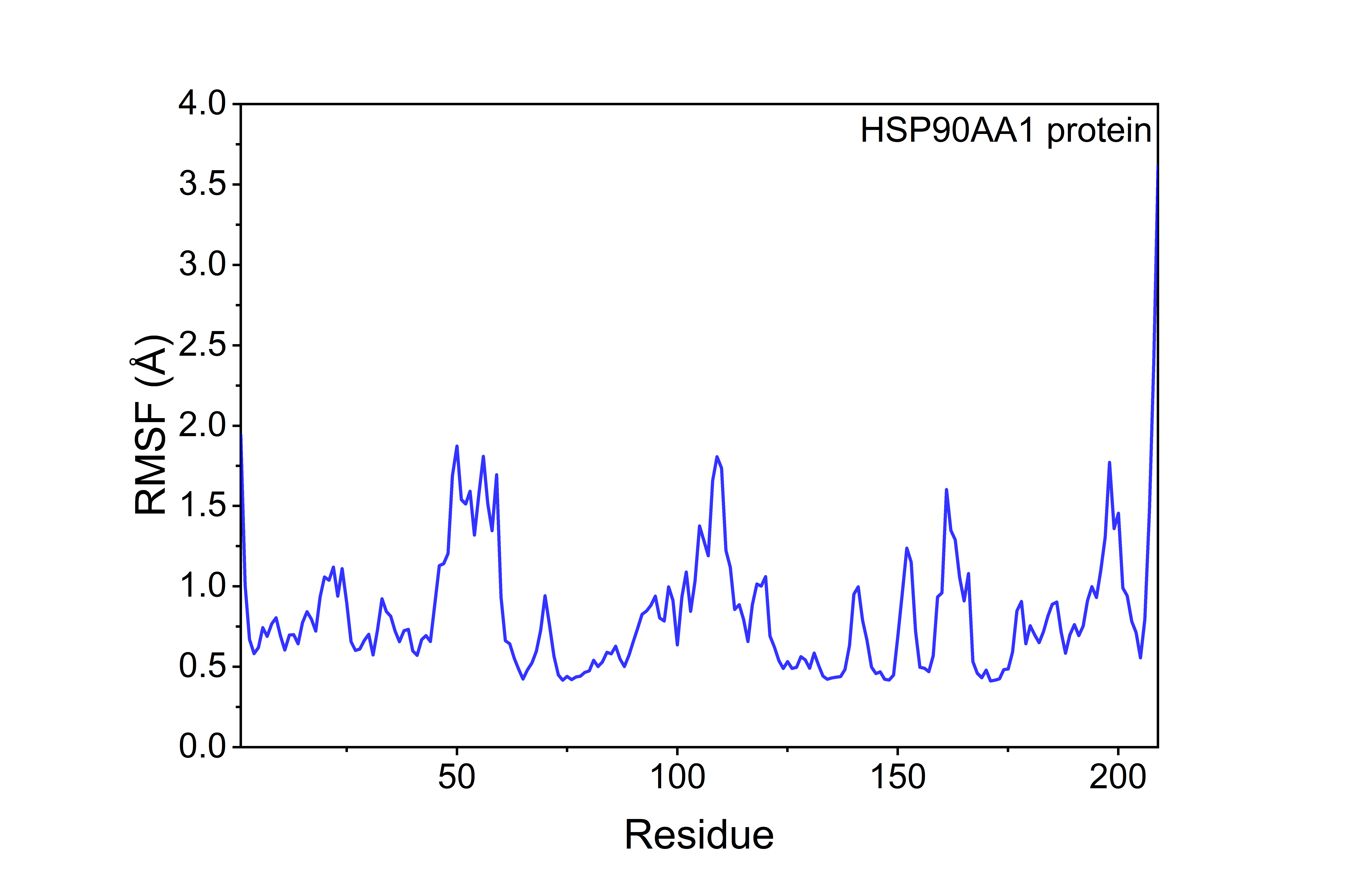

Supplement: Supplemental Information 7 [file peerj-10-13737-s007.zip › Molecular dynamics (MD) simulation data/HSP90AA1-Quercetin/RMSF-hsp90aa1-quercetin.tif]
