# Supplementary material for: Active ingredients and molecular targets of Taraxacum mongolicum against hepatocellular carcinoma: network pharmacology, molecular docking, and molecular dynamics simulation analysis
Source: PeerJ. 2022 Jul 18;10:e13737. doi: 10.7717/peerj.13737 (PMC9302432; doi:10.7717/peerj.13737)
Supplement: Supplemental Information 4 [file peerj-10-13737-s004.zip › Enrichment_GO/ColorByCluster.pdf]

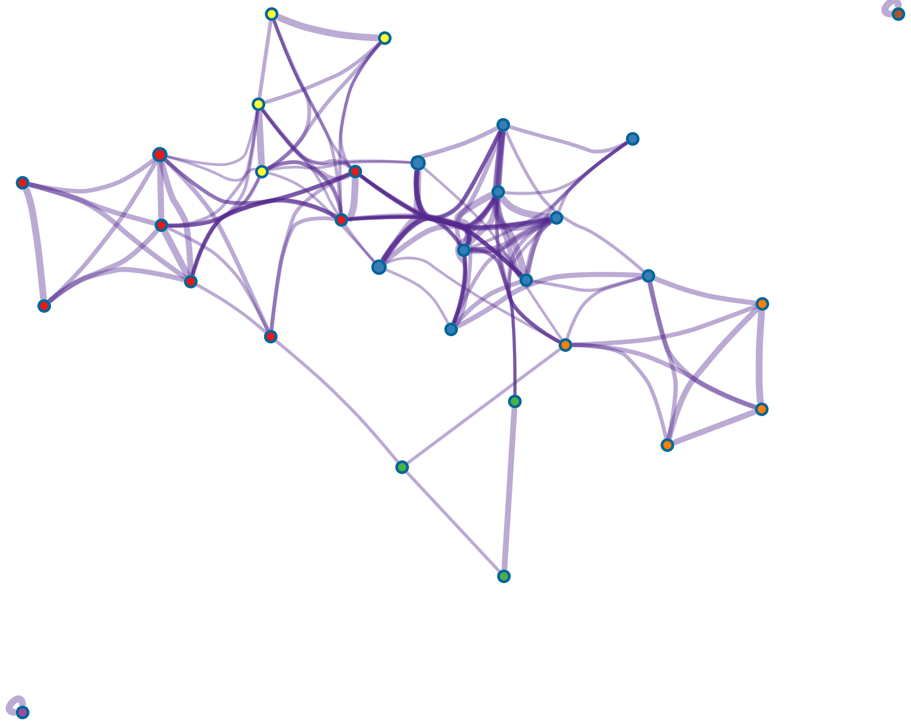

vesicle lumen
membrane raft
perinuclear region of cytoplasm
transcription regulator complex
cytoplasmic side of plasma membrane
dendrite
cell-cell junction

created by
http://metascape.org
