# Supplementary material for: Active ingredients and molecular targets of Taraxacum mongolicum against hepatocellular carcinoma: network pharmacology, molecular docking, and molecular dynamics simulation analysis
Source: PeerJ. 2022 Jul 18;10:e13737. doi: 10.7717/peerj.13737 (PMC9302432; doi:10.7717/peerj.13737)
Supplement: Supplemental Information 5 [file peerj-10-13737-s005.zip › Enrichment_GO/ColorByCluster.pdf]

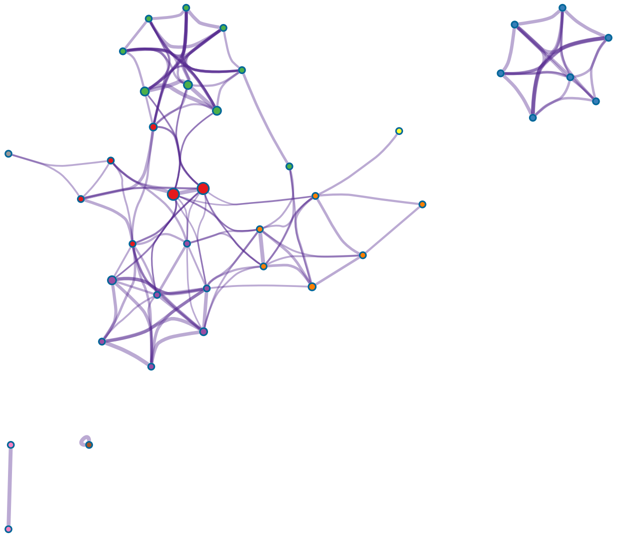

- protein kinase binding
- growth factor receptor binding
- protein kinase activity
- transcription factor binding
- ubiquitin protein ligase binding
- protease binding
- molecular function activator activity
- protein kinase regulator activity
- protein homodimerization activity

created by
http://metascape.org
