# Supplementary material for: Active ingredients and molecular targets of Taraxacum mongolicum against hepatocellular carcinoma: network pharmacology, molecular docking, and molecular dynamics simulation analysis
Source: PeerJ. 2022 Jul 18;10:e13737. doi: 10.7717/peerj.13737 (PMC9302432; doi:10.7717/peerj.13737)
Supplement: Supplemental Information 6 [file peerj-10-13737-s006.zip › AnalysisReport.pptx]

## Slide 1
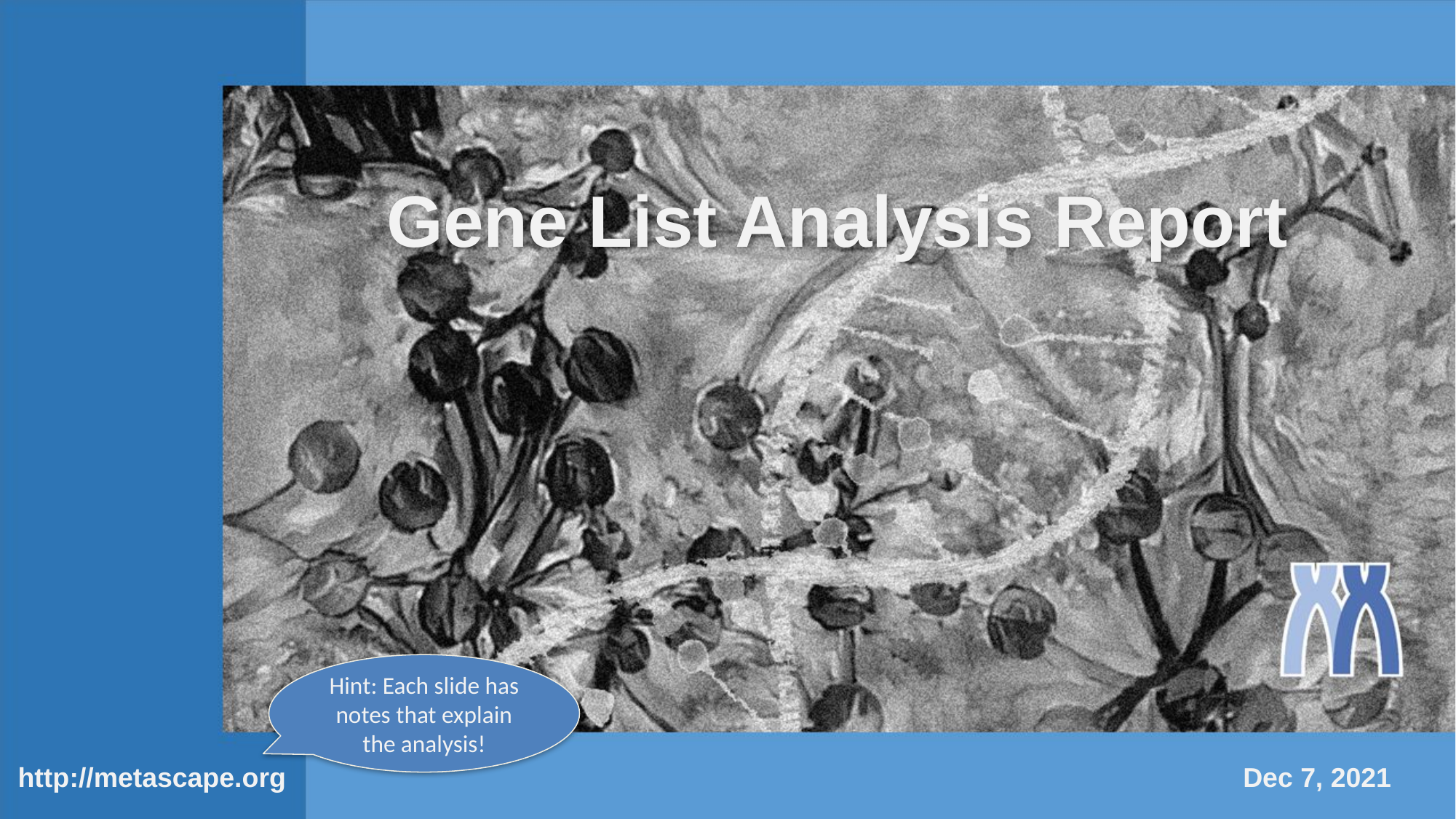

# Gene List Analysis Report
Hint: Each slide has notes that explain the analysis!
http://metascape.org
Dec 7, 2021

## Slide 2
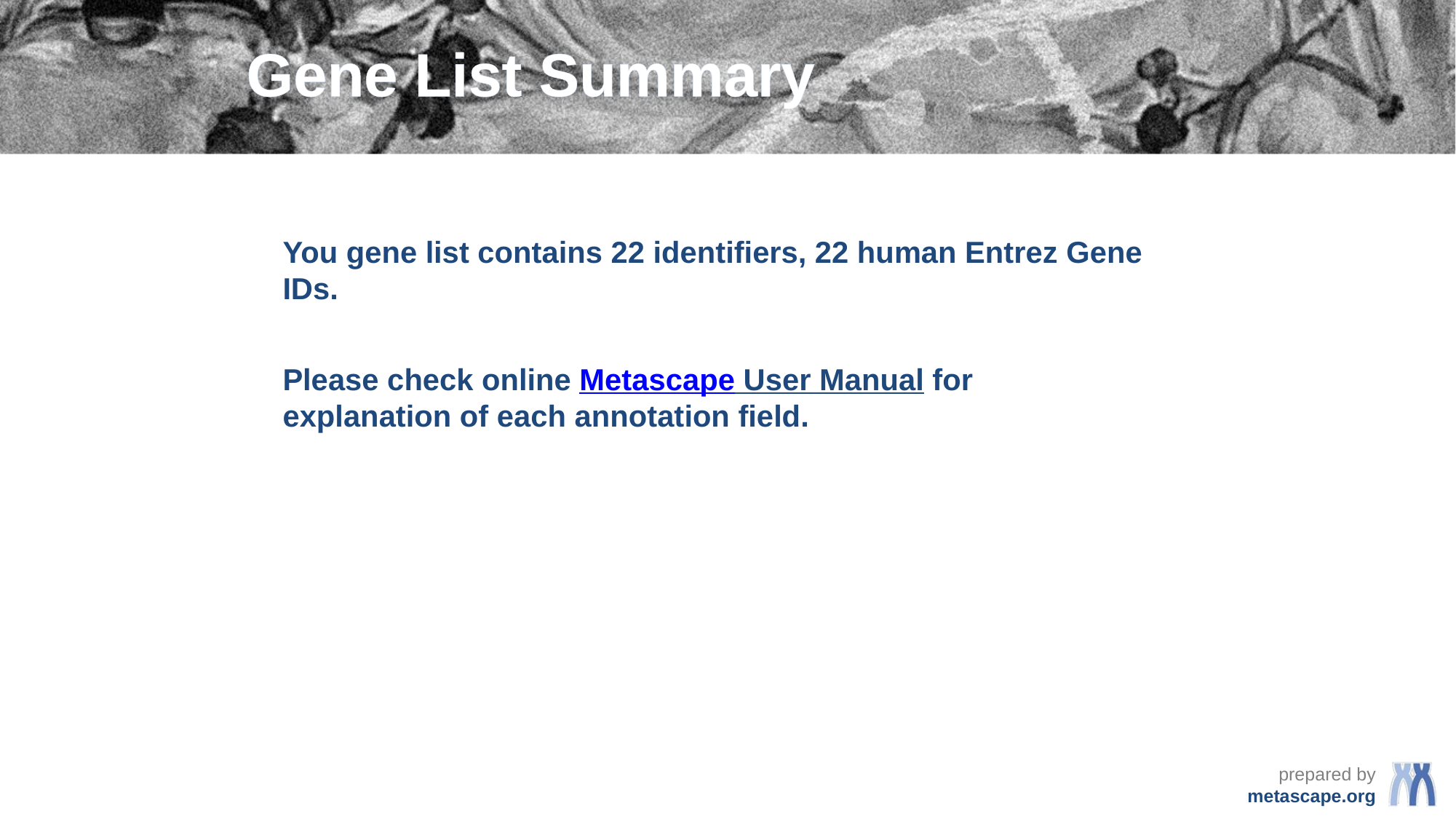

# Gene List Summary
You gene list contains 22 identifiers, 22 human Entrez Gene IDs.
Please check online Metascape User Manual for explanation of each annotation field.

## Slide 3
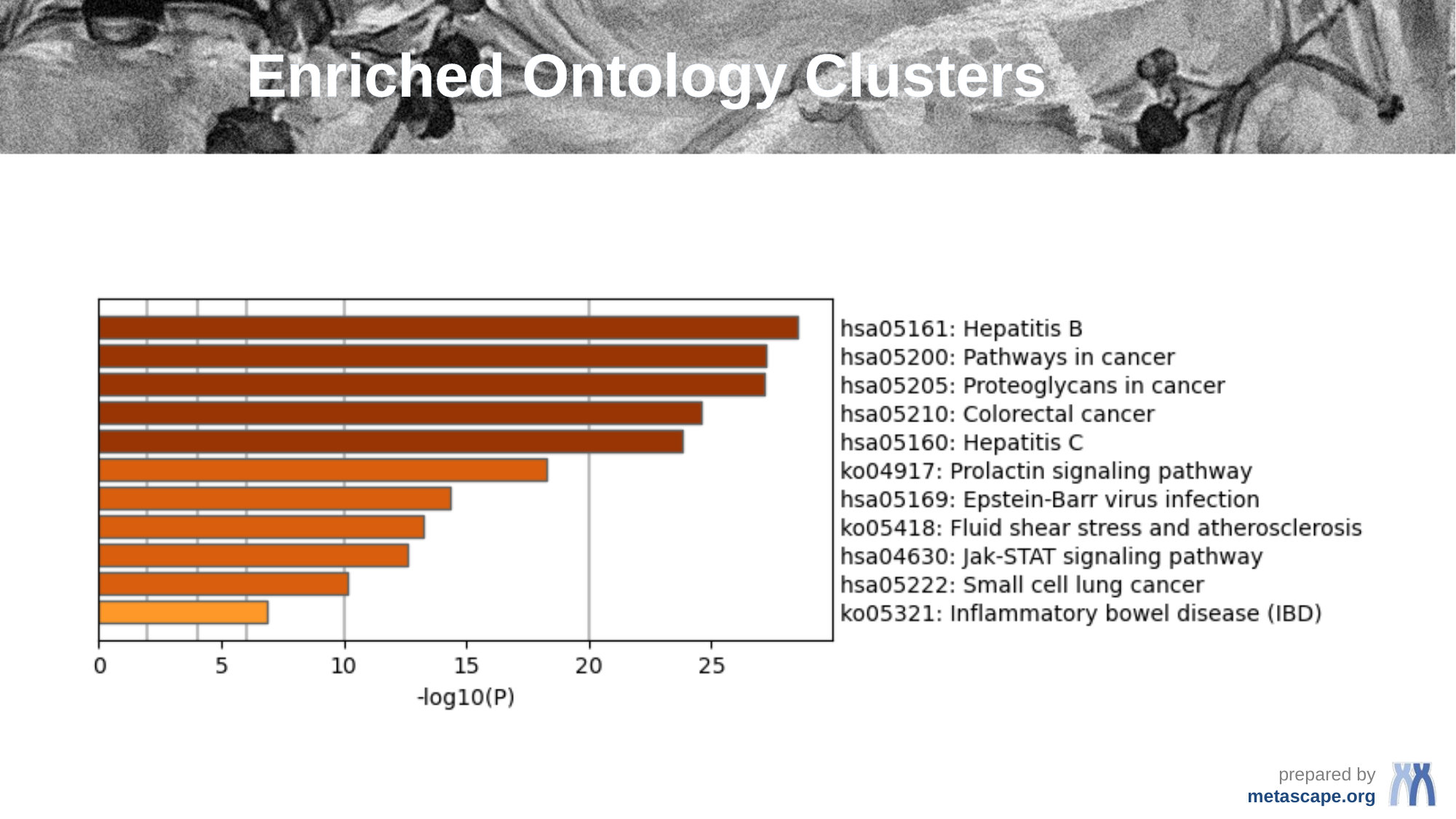

# Enriched Ontology Clusters

## Slide 4
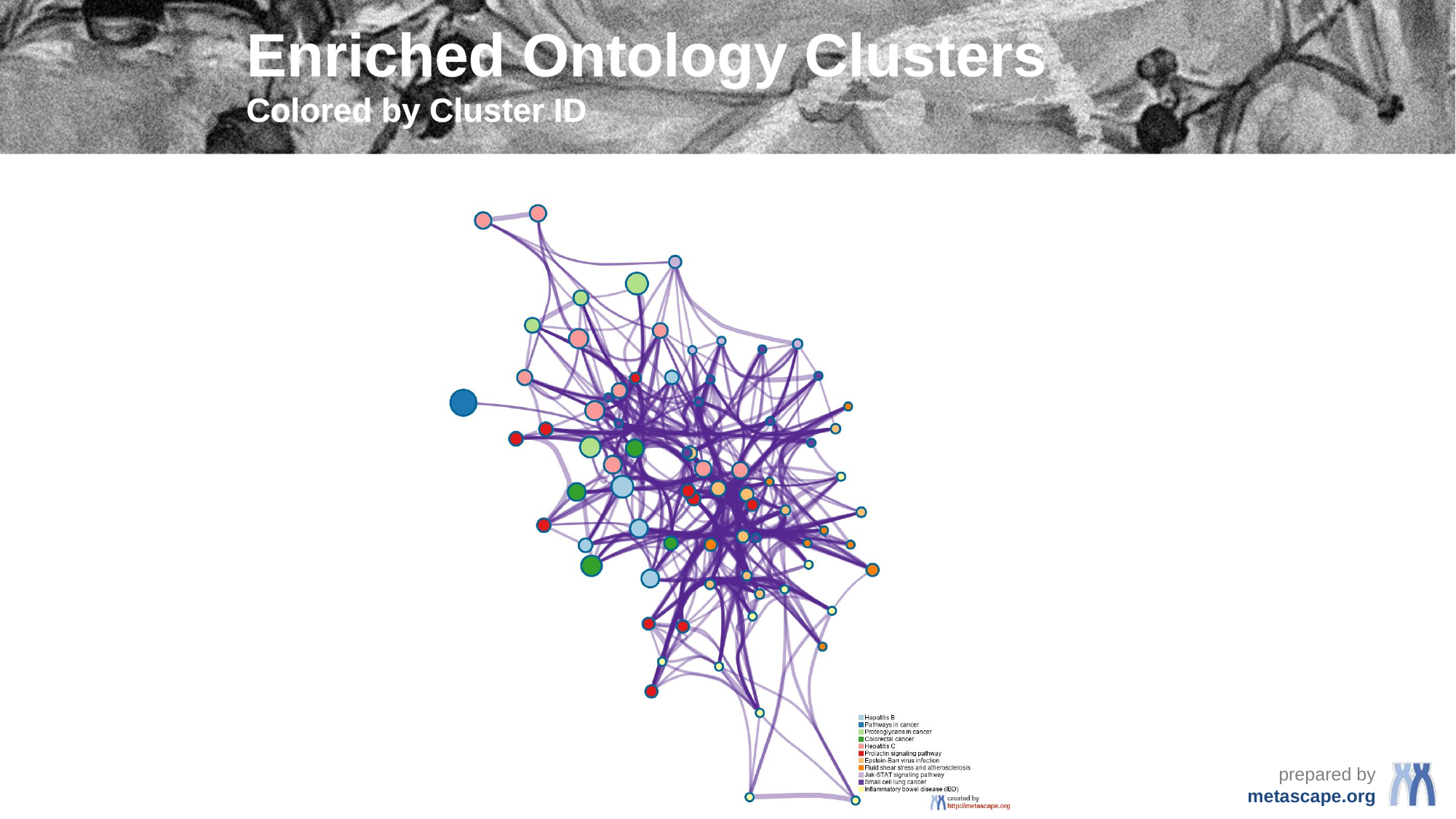

# Enriched Ontology Clusters Colored by Cluster ID

## Slide 5
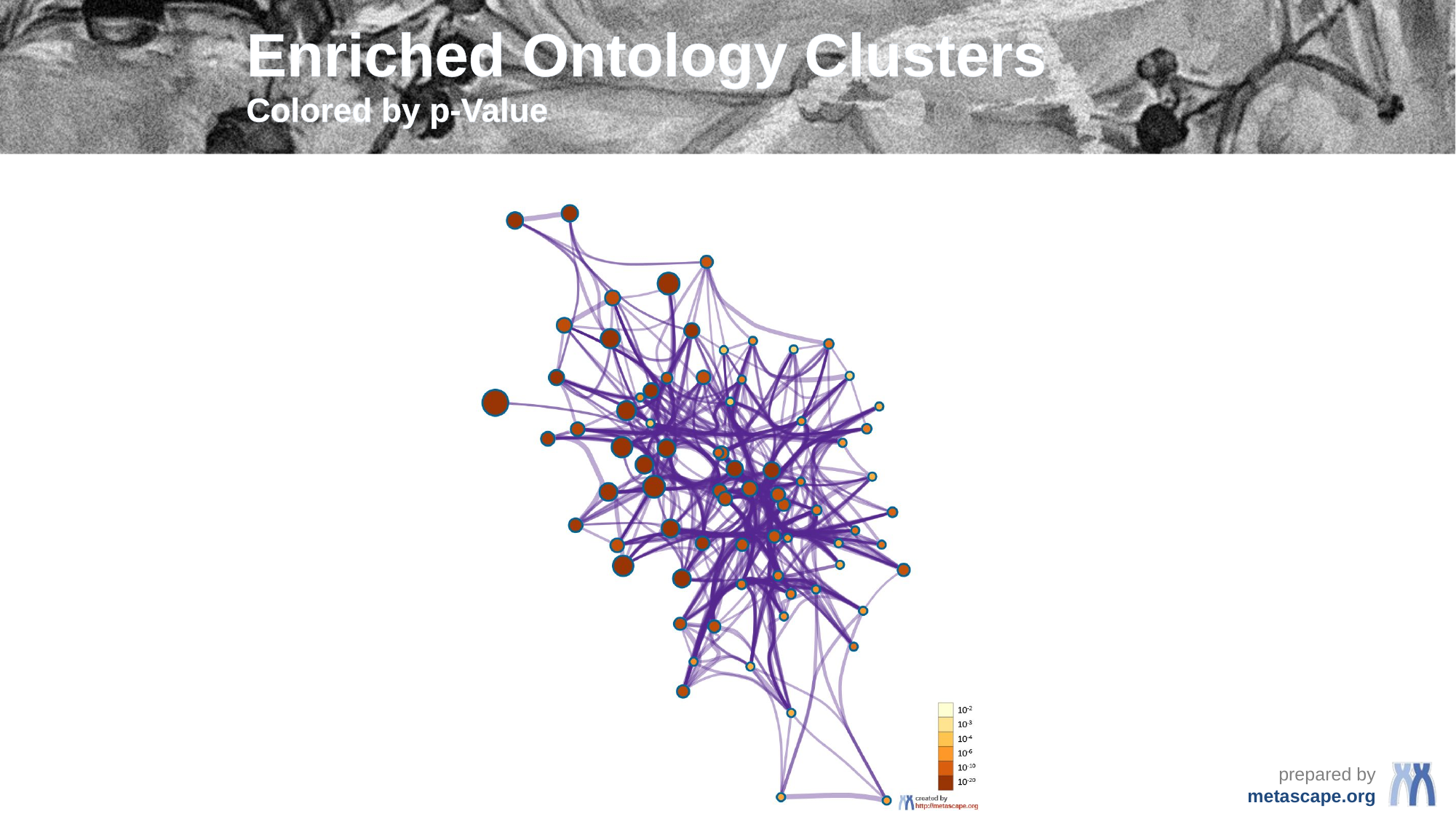

# Enriched Ontology Clusters Colored by p-Value

## Slide 6
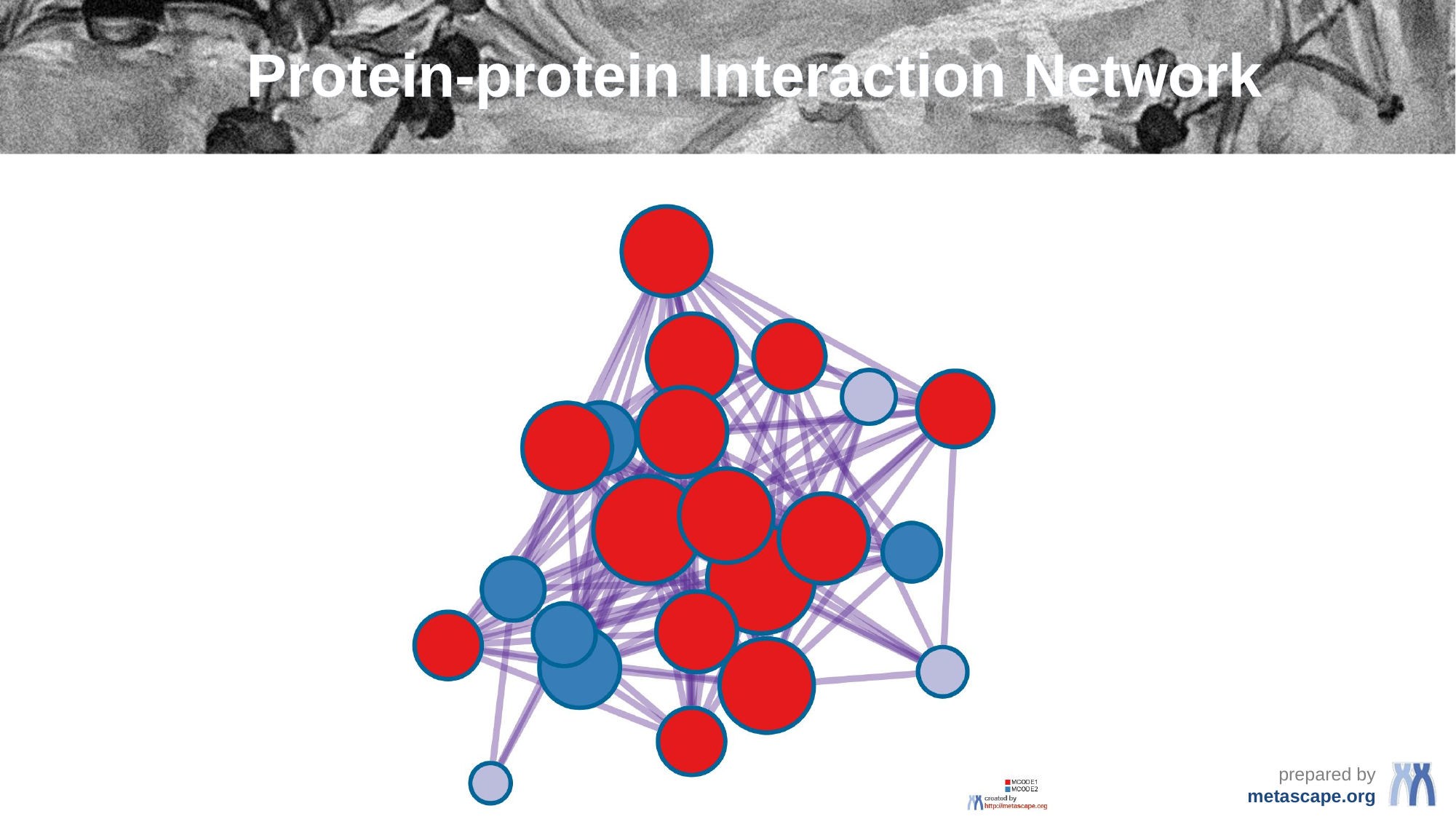

# Protein-protein Interaction Network

## Slide 7
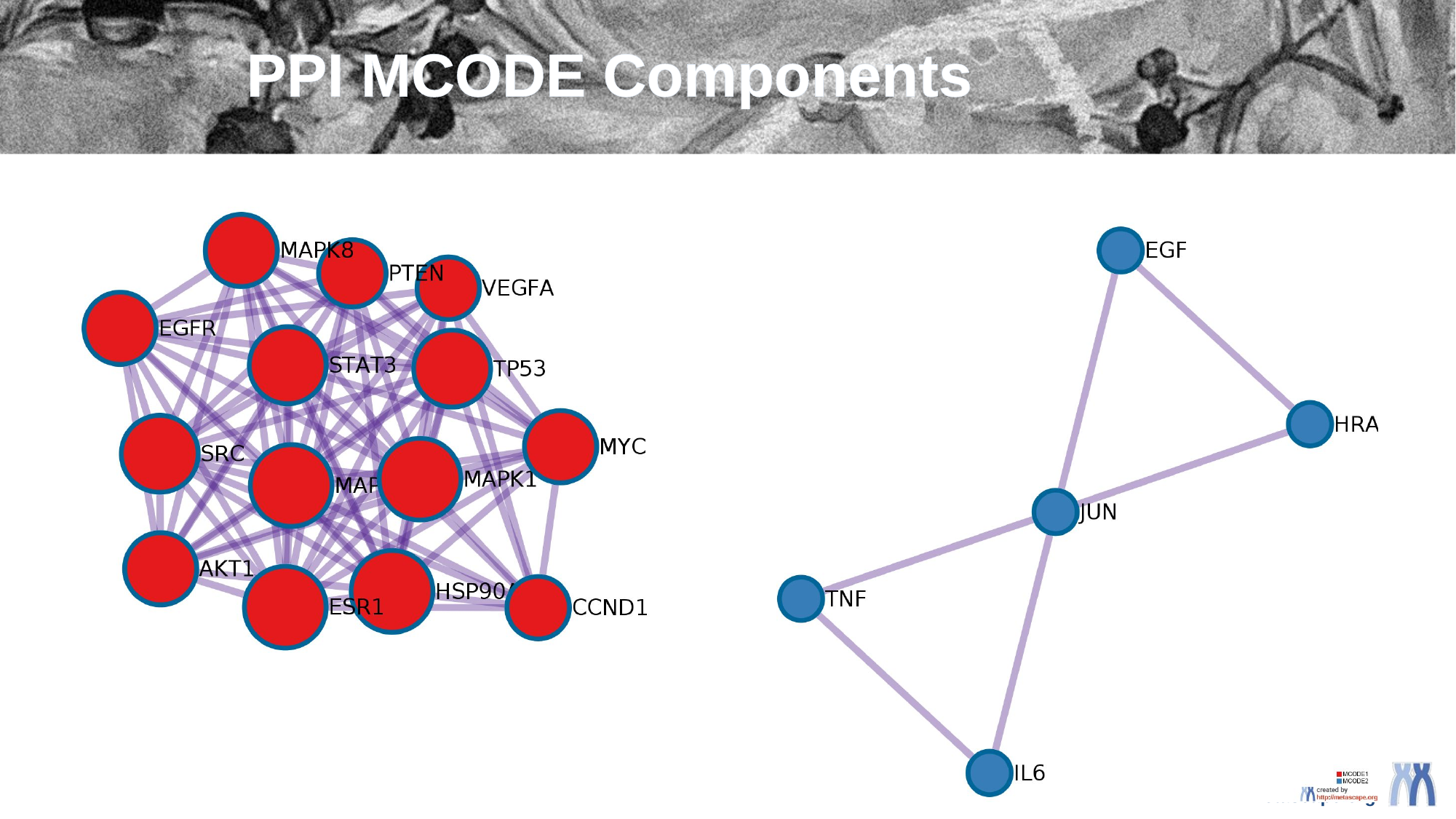

# PPI MCODE Components

## Slide 8
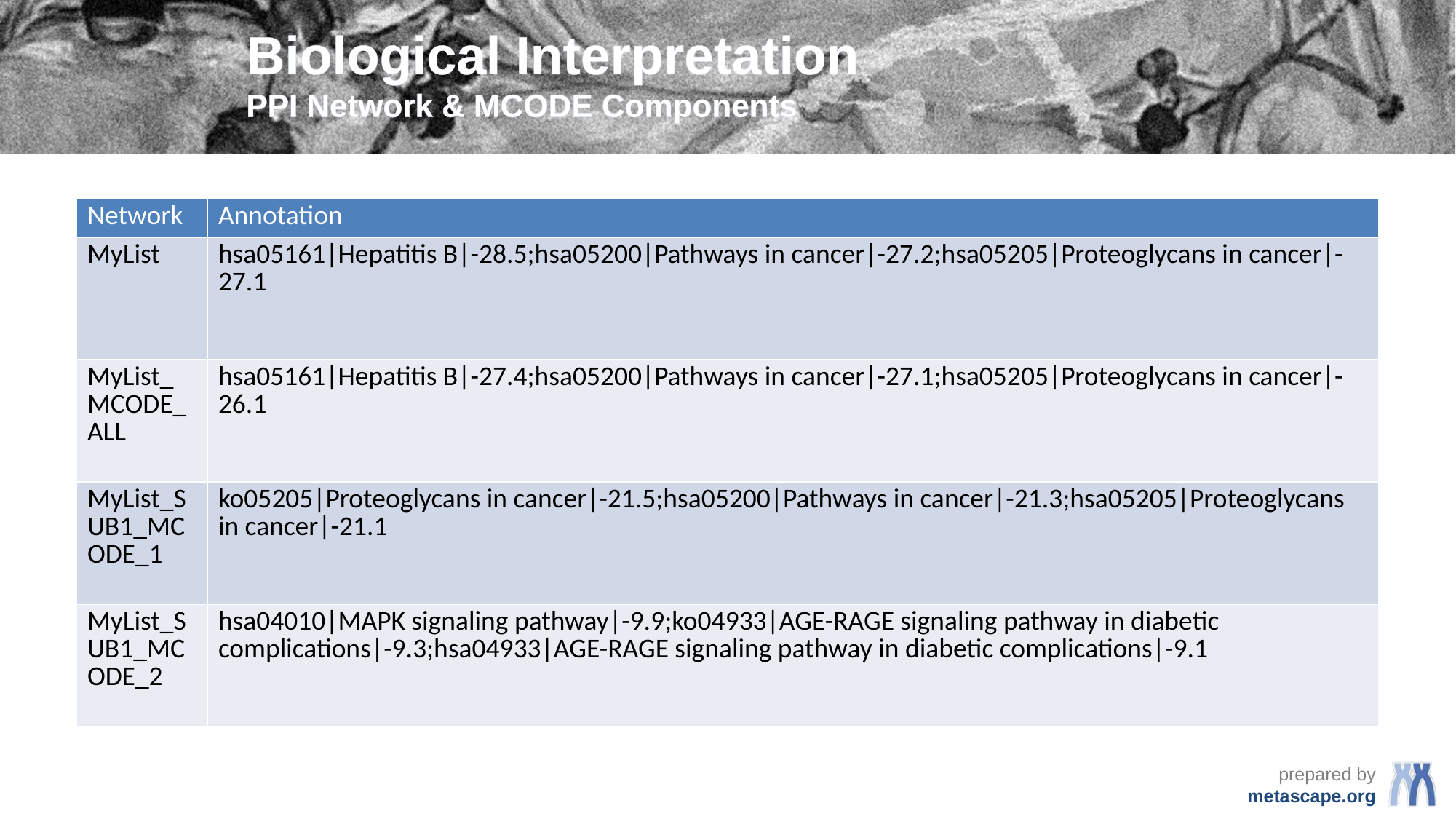

# Biological Interpretation PPI Network & MCODE Components
| Network | Annotation |
| --- | --- |
| MyList | hsa05161|Hepatitis B|-28.5;hsa05200|Pathways in cancer|-27.2;hsa05205|Proteoglycans in cancer|-27.1 |
| MyList\_MCODE\_ALL | hsa05161|Hepatitis B|-27.4;hsa05200|Pathways in cancer|-27.1;hsa05205|Proteoglycans in cancer|-26.1 |
| MyList\_SUB1\_MCODE\_1 | ko05205|Proteoglycans in cancer|-21.5;hsa05200|Pathways in cancer|-21.3;hsa05205|Proteoglycans in cancer|-21.1 |
| MyList\_SUB1\_MCODE\_2 | hsa04010|MAPK signaling pathway|-9.9;ko04933|AGE-RAGE signaling pathway in diabetic complications|-9.3;hsa04933|AGE-RAGE signaling pathway in diabetic complications|-9.1 |

## Slide 9
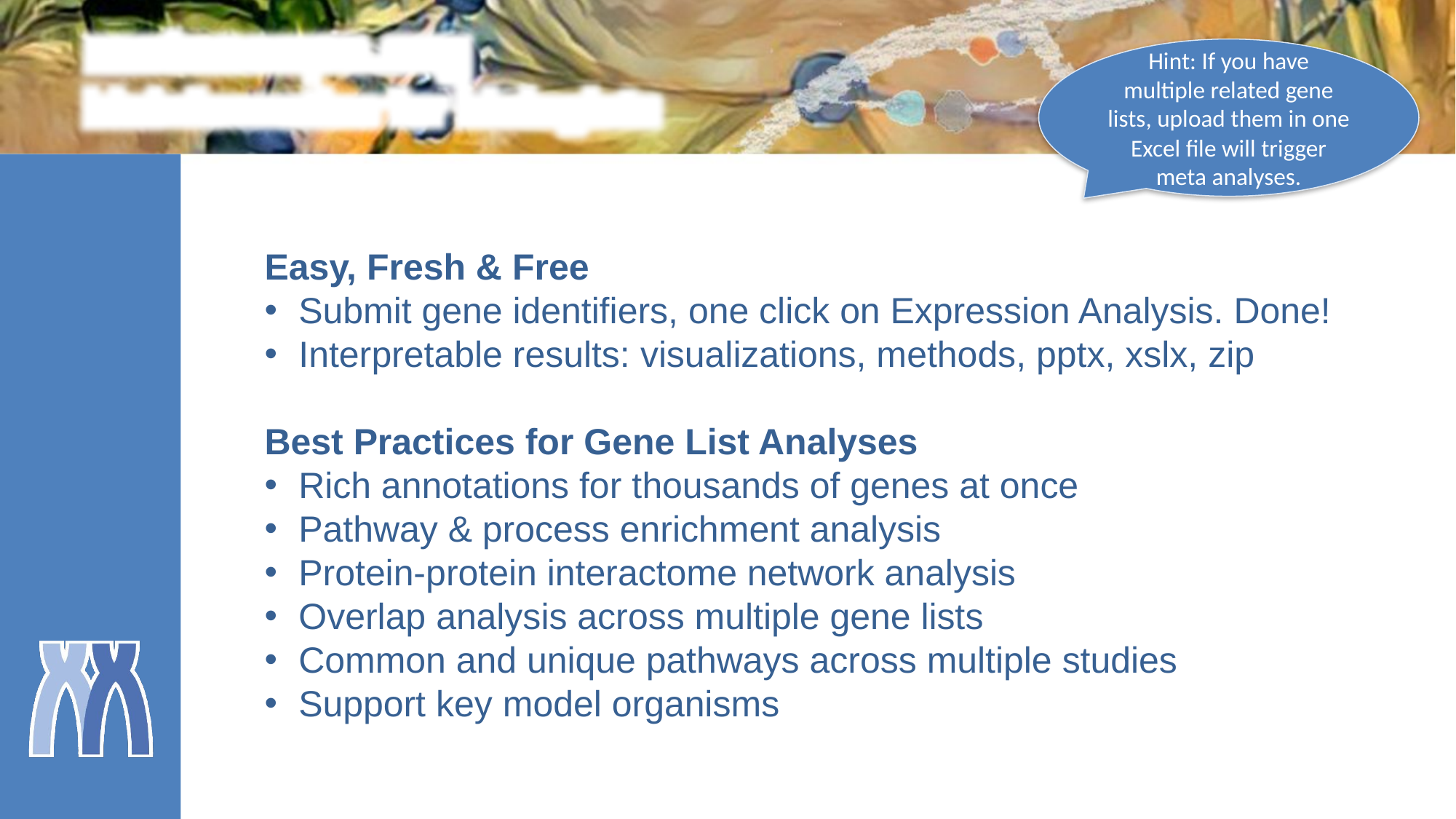

metascape.org
bioinformatics for biologists
Hint: If you have multiple related gene lists, upload them in one Excel file will trigger meta analyses.
Easy, Fresh & Free
Submit gene identifiers, one click on Expression Analysis. Done!
Interpretable results: visualizations, methods, pptx, xslx, zip
Best Practices for Gene List Analyses
Rich annotations for thousands of genes at once
Pathway & process enrichment analysis
Protein-protein interactome network analysis
Overlap analysis across multiple gene lists
Common and unique pathways across multiple studies
Support key model organisms
