# Supplementary material for: Active ingredients and molecular targets of Taraxacum mongolicum against hepatocellular carcinoma: network pharmacology, molecular docking, and molecular dynamics simulation analysis
Source: PeerJ. 2022 Jul 18;10:e13737. doi: 10.7717/peerj.13737 (PMC9302432; doi:10.7717/peerj.13737)
Supplement: Supplemental Information 6 [file peerj-10-13737-s006.zip › Enrichment_GO/ColorByCluster.pdf]

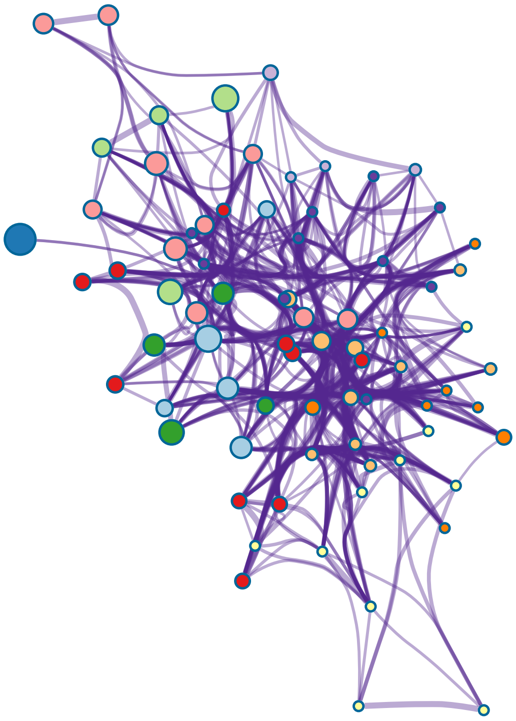

- Hepatitis B
- Pathways in cancer
- Proteoglycans in cancer
- Colorectal cancer
- Hepatitis C
- Prolactin signaling pathway
- Epstein-Barr virus infection
- Fluid shear stress and atherosclerosis
- Jak-STAT signaling pathway
- Small cell lung cancer
- Inflammatory bowel disease (IBD)

created by
http://metascape.org
